# Pan‐Cancer Analysis Links Altered RNA m^7^G Methyltransferase Expression to Oncogenic Pathways, Immune Cell Infiltrations and Overall Survival

**DOI:** 10.1002/cnr2.2138

**Published:** 2024-07-23

**Authors:** Anni Su, Renhua Song, Justin J.‐L. Wong

**Affiliations:** ^1^ Epigenetics and RNA Biology Laboratory, Charles Perkins Centre University of Sydney Camperdown Australia; ^2^ Faculty of Medicine and Health University of Sydney Camperdown Australia

## Abstract

**Background:**

N7‐methylguanosine (m^7^G) modification is one of the most prevalent RNA modifications in humans. Dysregulated m^7^G modifications caused by aberrant expression of m^7^G writers contribute to cancer progression and result in worse patient survival in several human cancers. However, studies that systematically assess the frequency and clinical relevance of aberrant m^7^G writer expression in a pan‐cancer cohort remain to be performed.

**Aims:**

This study aims to systematically investigate the molecular alteration and clinical relevance of m^7^G methyltransferase in human cancers.

**Methods:**

We analysed genome, transcriptome and clinical data from the Cancer Genome Atlas Research Network spanning 33 types of human cancers for aberrant changes in genes encoding m^7^G writers.

**Result:**

We demonstrate that m^7^G writers are dysregulated in human cancers and are associated predominantly with poorer survival. By dividing patients into those with high and low m^7^G scores, we show that a lower m^7^G score is generally associated with immune infiltration and better response to immunotherapy.

**Conclusion:**

Our analyses indicate the genetic alterations, expression patterns and clinical relevance of m^7^G writers across various cancers. This study provides insights into the potential utility of m^7^G writer expression as a cancer biomarker and proposes the possibility of targeting m^7^G writers for cancer therapy.

## 1 Introduction

N7‐methylguanosine (m^7^G) modification is one of the most prevalent RNA modifications in humans that regulates RNA metabolism [1]. m^7^G is deposited at 5′ cap and internal sites of messenger RNA (mRNA), transfer RNA (tRNA), ribosomal RNA (rRNA) and microRNA (miRNA) [1, 2]. So far, the understanding of m^7^G regulators remains preliminary. Several m^7^G writers have been identified. Methyltransferase like 1 (METTL1)/WD repeat domain 4 (WDR4) instals m^7^G on internal mRNA, tRNA and miRNA [3, 4]. The RNA guanine‐7 methyltransferase (RNMT)/family with sequence similarity 103 member A1 (FAM103A1) complex regulates the 5′ cap m^7^G modification [5]. Williams–Beuren syndrome chromosome region 22 protein (WBSCR22)/multifunctional methyltransferase subunit TRM112‐like protein (TRMT112) mediates rRNA m^7^G methylation [6]. However, the lack of reported m^7^G erasers and readers underscores a significant gap in the current knowledge [2, 7, 8]. Recently, the first internal m^7^G reader protein, quaking, has been identified [9]. This protein has been shown to regulate the stability and translational efficiency of a subset of internal m^7^G‐modified transcripts under stress conditions [9]. Although the understanding of m^7^G regulators is not sufficient, more and more evidence suggests that dysregulated m^7^G modification mediated by aberrant expression of m^7^G writers contributes to cancer development [1]. Overexpression of m^7^G writers METTL1/WDR4 results in an increased abundance of m^7^G‐methylated tRNA, which in turn upregulates the translational efficiency of cell cycle‐regulating transcripts to drive cancer progression [10]. In addition, METTL1/WDR4 regulates miRNA maturation in an m^7^G‐dependent manner, further contributing to cancer progression in lung and bladder cancer [4, 11]. Furthermore, another m^7^G writer WBSCR22 has been reported as an oncogene in glioma, as it promotes the proliferation, migration and invasion of glioma cells [12]. RNMT has been found to play an oncogenic role in PIK3CA mutant breast cancer [13]. These findings highlight the importance of understanding the role of m^7^G writers in cancer and their potential implications for therapy. However, few studies have comprehensively evaluated the genetic alteration and clinical relevance of m^7^G writers in pan‐cancer cohorts. Here, we systematically investigated the molecular alteration and clinical relevance of m^7^G writers across 33 human cancers.

## 2 Methods

### 2.1 Data Collection and Processing

All datasets including gene expression, clinical characteristics, somatic mutation and copy number alteration were downloaded from the Cancer Genome Atlas (TCGA, https://portal.gdc.cancer.gov/). The m^7^G writers (METTL1, WDR4, RNMT, FAM103A1, WBSCR22 and TRMT112) were sourced from the previous literature [1]. All data processing steps and statistical analyses were performed in the R v4.2.2 statistical environment (http://www.r‐project.org).

### 2.2 Description of 33 Types of Cancer From TCGA

In this study 33 different types of cancer from TCGA were analysed, including ACC, adrenocortical carcinoma (*n* = 79 primary tumour [TP]); BRCA, breast cancer (*n* = 113 normal tissue [NT], 1111 TP); BLCA, bladder urothelial carcinoma (*n* = 19 NT, 412 TP); CESC, cervical squamous cell carcinoma and endocervical adenocarcinoma (*n* = 3 NT, 304 TP); CHOL, cholangiocarcinoma (*n* = 9 NT, 35 TP); COAD, colon adenocarcinoma (*n* = 41 NT, 481 TP); DLBC, lymphoid neoplasm diffuse large B‐cell lymphoma (*n* = 48 TP); ESCA, oesophageal carcinoma (*n* = 13 NT, 184 TP); GBM, glioblastoma multiforme (*n* = 5 NT, 157 TP); HNSC, head and neck squamous carcinoma (*n* = 44 NT, 520 TP); KICH, kidney chromophobe (*n* = 25 NT, 66 TP); KIRC, kidney renal clear cell carcinoma (*n* = 72 NT, 541 TP); KIRP, kidney renal papillary cell carcinoma (*n* = 32 NT, 290 TP); LAML, acute myeloid leukaemia (*n* = 151 TP); LGG, brain lower grade glioma (*n* = 516 TP); LIHC, liver hepatocellular carcinoma (*n* = 50 NT, 371 TP); LUAD, lung adenocarcinoma (*n* = 59 NT, 539 TP); LUSC, lung squamous cell carcinoma (*n* = 51 NT, 502 TP); MESO, mesothelioma (*n* = 87 TP); OV, ovarian serous cystadenocarcinoma (*n* = 422 TP); PAAD, pancreatic adenocarcinoma (*n* = 4 NT, 178 TP); PCPG, pheochromocytoma and paraganglioma (*n* = 3 NT, 179 TP); PRAD, prostate adenocarcinoma (*n* = 51 NT, 501 TP); READ, rectum adenocarcinoma (*n* = 10 NT, 166 TP); SARC, sarcoma (*n* = 2 NT, 259 TP); SKCM, skin cutaneous melanoma (*n* = 1NT, 103TP); STAD, stomach adenocarcinoma (*n* = 36 NT, 412 TP); TGCT, testicular germ cell tumours (*n* = 150 TP); THCA, thyroid carcinoma (*n* = 59 NT, 505 TP); THYM, thymoma (*n* = 2 NT, 120 TP); UCEC, uterine corpus endometrial carcinoma(*n* = 25 NT, 553 TP); UCS, uterine carcinosarcoma (*n* = 57 TP) and UVM, uveal melanoma (*n* = 80 TP).

### 2.3 Analysis of Copy Number Alteration and Somatic Mutation Frequency

CNA data for patients of 33 cancer types were downloaded from TCGA Gistic2 Level 4 datasets via getData() R‐package ‘RTCGAToolbox’ from Broad GDAC Firehose (https://gdac.broadinstitute.org/) [14]. Genes with copy number >2 were defined as amplified and genes with copy number <−2 were defined as deleted.

The mutation data for patients of 33 cancer types were downloaded from TCGA Gistic2 Level 3 data sets via getData() R‐package ‘RTCGAToolbox’ with mutation = TRUE.

### 2.4 Analysis of Differential Gene Expression Across Cancer Types

The RNA‐seq dataset, Fragments per kilobase of transcript per million mapped reads upper quartile (FPKM‐UQ)‐based gene expression for 33 types of cancer were obtained from TCGA via the R package ‘TCGAbiolinks’. The log2 fold‐change was calculated based on the formula.

$$\log2\text{fold}‐\text{change}=\log2\left(\frac{\text{Mean}\;\text{expression}\;\text{tumour}\;\text{tissue}}{\text{Mean}\;\text{expression}\;\text{normal}\;\text{tissue}}\right).$$

Comparisons of expression data between tumour and normal tissues were conducted only in cancers with at least five tumour‐normal pairs. The statistical differences are calculated by using the Wilcoxon rank‐sum test. *p* value <0.05 was considered statistically significant.

The expression correlations among *METTL1*, *WDR4*, *RNMT*, *FAM103A1*, *WBSCR22* and *TRMT112* among TCGA tumours were conducted by using the Pearson correlation coefficient. The protein–protein interaction among m^7^G writers is based on the STRING interaction database [15].

### 2.5 Survival Analysis

The clinical information for patients of 33 cancers type was downloaded via ‘TCGAbiolinks’ including vital status, days to death and days to last follow‐up. The prognosis of patients' overall survival (OS) was compared based on the median expression levels of each m^7^G writer. The log‐rank test was used to test the different OS rates between two groups by R‐package ‘survival’ (https://CRAN.R‐project.org/package=survival). Kaplan–Meier curves were generated to visualise the differences in the OS of patients by ‘survminer’ R‐packages (https://rpkgs.datanovia.com/survminer/index.html). The univariate and multivariate Cox proportional hazards regression models for different cancers were analysed by the ‘survival’ and ‘survminer’ R‐packages. *p* value <0.05 were considered significant.

### 2.6 Analysis of Correlation Between m^7^G Writer Gene Expression and Hallmark Pathway

Hallmark gene sets were obtained from MSigDB. Gene Set Variation Analysis (GSVA) was performed to elucidate the underlying biological pathway and function differences associated with the altered gene signature [16]. GSVA was performed using the ‘GSVA’ R package. To identify the m^7^G writers that were correlated with activation or inhibition of a cancer‐related‐hallmark pathway, the Pearson Correlation Coefficient (PCC) between the expression of m^7^G writers and pathway activity was calculated by ‘Hmisc’ R‐package (https://cran.r‐project.org/web/packages/Hmisc/index.html). The |PCC| >0.5 and adjusted *p* value <0.05 were set as thresholds to identify a significant correlation between m^7^G writers and hallmark‐related pathways.

### 2.7 m^7^G Score

To quantify the m^7^G modification pattern in each cancer patient, the m^7^G scoring system was generated by principal component analysis (PCA). Principal components 1 and 2 were used as scoring coefficients. The following formula was used to calculate the score for each sample, where *i* represents the expression of the m^7^G writer gene.

$$m^{7}G\;\text{score}=\sum \left(\mathrm{PC}1i+\mathrm{PC}2i\right).$$

The patients were divided into high and low m^7^G score groups by ‘surMisc’ R‐package (https://cran.r‐project.org/web/packages/survMisc/index.html). To investigate the prognostic significance of the m^7^G score, Kaplan–Meier analysis was performed on the high and low m^7^G score group with the ‘survival’ and ‘survminer’ R package.

### 2.8 Immune Cell Infiltration

Tumour microenvironment immune cell infiltration was determined by single‐sample Gene Set Enrichment analysis (ssGESA). The 46 integrated immune gene sets were obtained from the published studies [17]. The ssGSEA scores of each gene were calculated across 10 079 samples in the pan‐cancer cohort. The correlation between immune cells and m^7^G writers' expression was calculated by Pearson's method by ‘Hmisc’ R‐package.

The CIBERSORT algorithm is a method to characterise the cell composition of tumour tissues based on their gene expression profiles [18]. LM22 is an expression data for 22 common immune cells obtained from CIBERSORT. The proportion of 22 immune cells in the high and low m^7^G expression group was calculated by CIBERSORT.

## 3 Result

**FIGURE 1:**
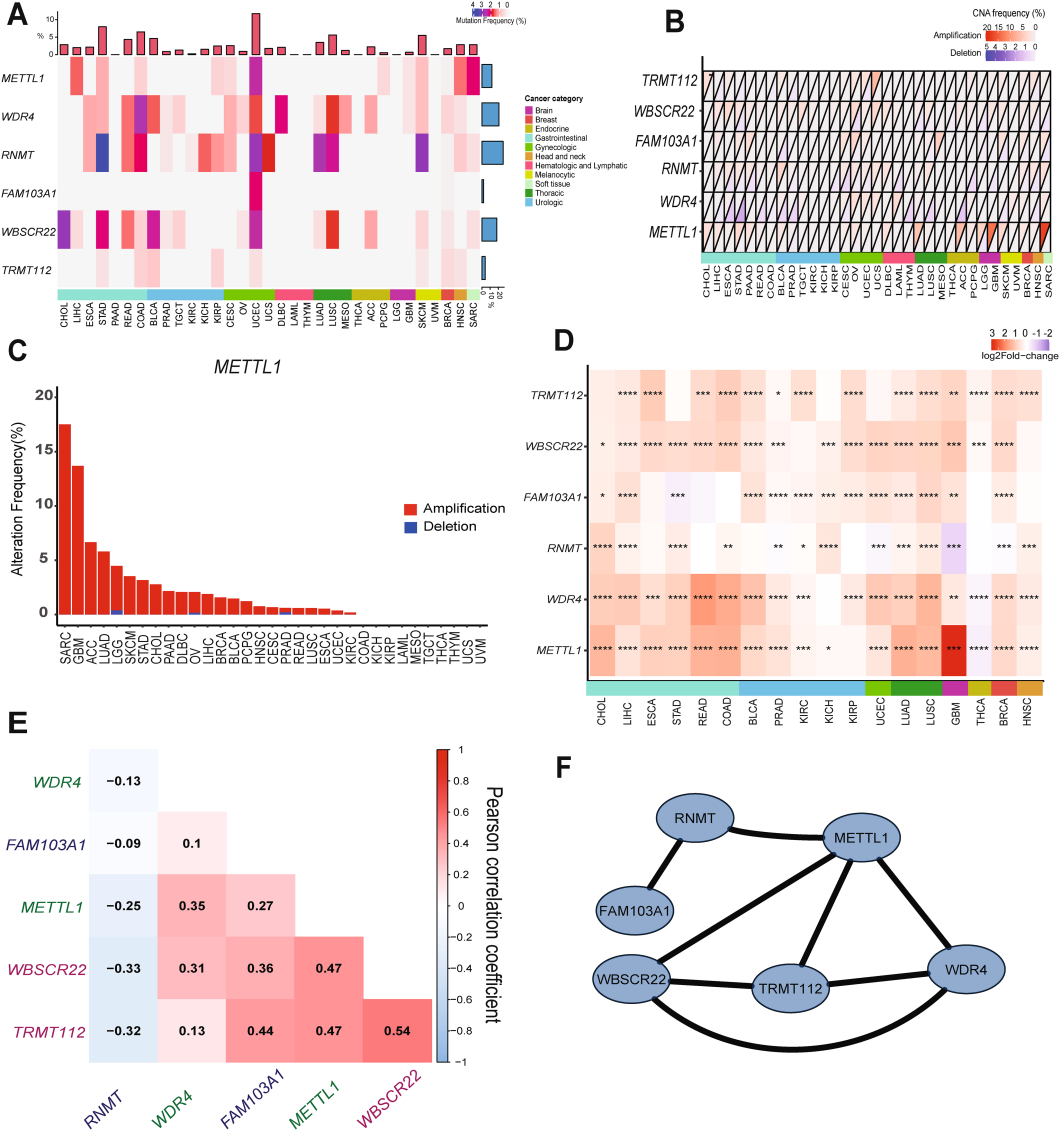
Genetic alteration and expression changes of m^7^G writers in 33 human cancers. (A) Mutation frequency of m^7^G writers across 33 cancer types. The upper bar plot indicates the overall m^7^G mutation frequency in individual cancers. The right bar plot represents the overall m^7^G mutation frequency in 33 cancer types. A total of 33 types of human cancer are categorised based on their sites of occurrence and indicated by the same colour in the annotated bar. (B) The CNA alteration frequency of m^7^G writer genes across 33 cancer types. The upper part of each grid represents amplification frequency and the lower part represents the frequency of deletion. (C) Copy number alteration frequency of *METTL1* in 33 cancer types. The red and blue bars represent the frequency of amplification and deletion, respectively. (D) Differential gene expression of m^7^G writer genes in 18 cancer types. The heatmap represents log2 fold‐change (tumour/normal) with red and blue representing upregulated and downregulated genes, respectively. For (B and D), the site of occurrence for each cancer is annotated in the coloured bar as in (A). The Wilcoxon rank test was used to compare the two groups. Significance was denoted by *p < 0.05; **p < 0.01; ***p < 0.001 and ****p < 0.0001. (E) Gene expression correlation between the m^7^G writers. Genes that belong to the same m^7^G writer complex are printed in the same colour. (F) The protein–protein interaction map of m^7^G writers.

We first investigated the somatic mutation of m^7^G writers in cancers. Although the average mutation frequency of m^7^G writers was low (0.1%–3.5%), cancers with higher global mutation burden [19], including UCEC, STAD and SKCM, exhibited a higher mutation frequency of m^7^G writers (Figure 1A and Table S1). There was a uniquely higher mutation frequency of *RNMT* in different cancer types, particularly in UCEC, STAD, SKCM and LUAD (Figure 1A).

We then determined the frequency of copy number alteration (CNA) in m^7^G writer genes across 33 cancer types. We observed a generally low frequency of amplification and deletion in m^7^G writer genes across 33 cancer types (<10%) (Figures 1B and S1; Table S2), apart from *METTL1*, which showed amplification across various cancer types, particularly in SARC and GBM (~15%) (Figure 1B,C).

We explored the expression patterns of m^7^G writers in cancers by comparing their expression levels between primary tumours and normal tissues in 18 cancer types. We observed an overall higher expression of m^7^G writers in tumours compared with normal tissues regardless of the site of origin (Figures 1D, S2 and S3). Notably, lung cancer (LUSC and LUAD) exhibited upregulation of all m^7^G writers, implying a potential role of m^7^G modification in lung cancer. Consistent with the prevalence of *METTL1* amplification, *METTL1* was significantly upregulated in GBM tumours (Figure 1D). Notably, *METTL1* and *WDR4* are downregulated in cancers that originate from the endocrine system (Figures 1D and S3). For the m^7^G cap writers, we observed an opposing expression pattern of *RNMT* and *FAM103A1* in cancer (Figure 1D). *RNMT* was downregulated in GBM and UCEC tumours, consistent with the overall downregulation of *RNMT* in brain and gynaecological cancers (Figure S3), whereas the expression of *FAM103A1* increased in these cancers (Figure 1D). Our results demonstrated that m^7^G writers were dysregulated in human cancer indicating their potential roles in tumourigenesis.

We further investigated the correlation of expression levels between m^7^G writer genes. In general, there was a positive correlation among writers' genes that belonged to the same protein complex, except *RNMT*. Notably, *RNMT* negatively correlated with all other m^7^G writers including its cofactor, *FAM103A1* (Figure 1E). m^7^G writers interacted with each other based on the protein–protein interaction network (Figure 1F). Overall, our result indicates a synergistic expression of *METTL1*/*WDR4* genes in cancers, consistent with their functions as a complex. This observation holds true for *WBSCR22/TRMT112* genes that form another m^7^G writer complex targeting rRNA. The negative correlation of expression levels between *RNMT* and *FAM103A1* is surprising given their role as a complex that regulates 5′ cap m^7^G. It is conceivable that FAM103A1 may have another binding partner that acts in place of RNMT. RNMT may also have a role in tumourigenesis that is independent of FAM103A1. Our data provides information regarding the types of cancers that may be suitable for future investigations into these possibilities (Figure 1D).

**FIGURE 2:**
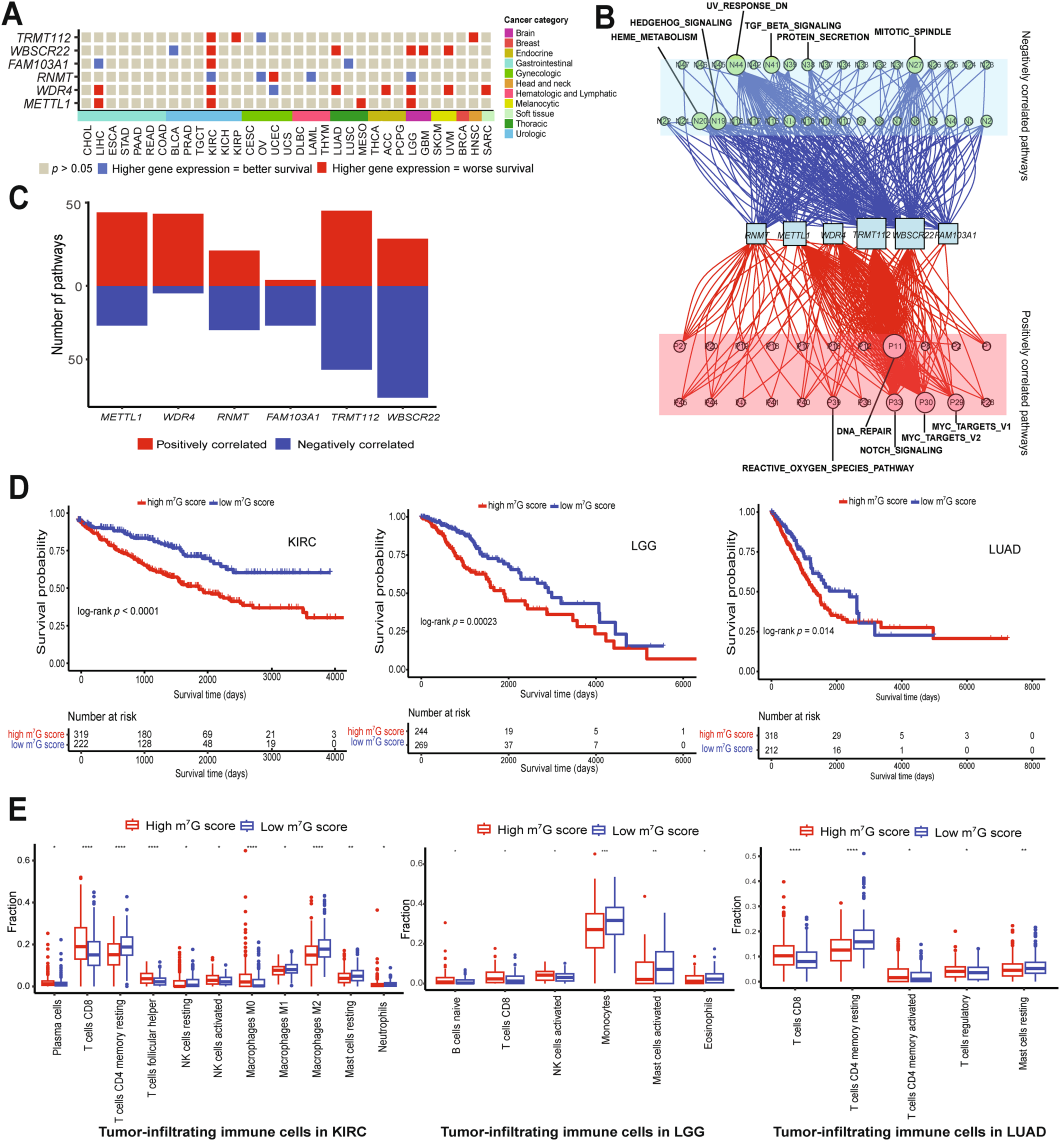
Clinical relevance of m^7^G writers in human cancer. (A) An Association between expression of m^7^G writers and overall patient survival in 33 cancer types. Red represents higher gene expression associated with worse overall survival. Blue represents higher gene expression associated with better overall survival. A total of 33 types of human cancer are categorised based on their sites of occurrence and indicated by the same colour in the annotated colour bar. (B) The network diagram indicates the correlation between m^7^G writers and cancer pathways. Red nodes show positively‐correlated pathways and green nodes show negatively‐correlated pathways. The size of nodes represents the number of links. (C) The number of pathways correlated with individual m^7^G writers. The upper red panel shows positively correlated pathways, and the bottom blue panel shows negatively correlated pathways. (D) Kaplan–Meier survival plot in KIRC (left), LGG (middle) and LUAD (right) grouped by m^7^G score. (E) Box plot shows immune cell infiltration composition of tumour microenvironment in m^7^G‐high and ‐low groups in KIRC (left), LGG (middle) and LUAD (right). Red and blue represent the m^7^G‐high and ‐low groups. The Wilcoxon rank test was used to compare the two groups. Significance was denoted by *p < 0.05; **p < 0.01; ***p < 0.001 and ****p < 0.0001.

When examining clinical relevance, our findings revealed that higher expression of m^7^G writers was associated with the overall survival of patients in 15 cancer types (Figures 2A and S4). Higher expression of m^7^G writers, *METTL1*, *WDR4*, *FAM103A1*, *WBSCR22* and *TRMT112*, was significantly associated with poor overall survival of KIRC patients. In contrast, *RNMT* overexpression conferred better overall survival in KIRC (Figure 2A and S5). Higher expression of *METTL1* and its cofactor *WDR4* were significantly associated with poorer overall survival in LGG and LIHC, indicating an oncogenic role of the METTL1/WDR4 methyltransferase in these cancers (Figure 2A). *WBSCR22* displayed potential oncogenic features in brain cancers (LGG and GBM). Higher expression of *TRMT112* was associated with poorer overall survival in kidney cancers (KIRC and KIRP) (Figure 2A). Collectively, these results suggest that m^7^G writers have the potential to predict survival outcomes in a cancer‐type‐specific manner.

We investigated the molecular mechanisms by which m^7^G writers contribute to cancer pathogenesis by correlating the expression of m^7^G writers with the activity of 50 cancer hallmark‐related pathways. The expression of m^7^G writers was significantly correlated with the activation of pathways including DNA repair, MYC and Notch signalling (Figure 2B and Table S3). Conversely, m^7^G writer expression correlated with the inhibition of pathways, including downregulation of genes under UV radiation, mitotic spindle assembly, TGF beta signalling pathways and heme metabolism (Figure 2B and Table S3). Furthermore, the expression of *WBSCR22* and *TRMT112* showed correlations with a higher number of both activated and inhibited pathways (Figure 2C). Our data indicate that m^7^G writers may play a role in the activation and inhibition of cancer‐related pathways.

We further explored the infiltration of immune cells in cancers based on the overall expression of m^7^G writers. Given that our analysis revealed higher expression of nearly all m^7^G writers in KIRC, LGG and LUAD patients with poorer overall survival (Figure 2A) and emerging evidence that immunotherapy improves prognosis in KIRC, LGG and LUAD patients [20, 21, 22], we analysed immune cell infiltration in KIRC, LGG and LUAD patients. We divided these into two groups: the m^7^G‐high group and the m^7^G‐low score group. We observed that patients with high m^7^G scores showed poor prognosis (Figure 2D). Furthermore, we performed multivariate Cox regression analysis including the m^7^G score and main clinicopathological features (gender, age, race, grade, stage, tumour, node and metastasis). We subsequently stratified these main clinicopathologic features to investigate whether the m^7^G score affects prognosis within these groups (Figures [Link], [Link]). The analysis showed that the m^7^G score was significantly associated with poor prognosis in KIRC, LGG and LUAD, suggesting it is an independent prognostic factor in these cancers. This indicates that the m^7^G score is an effective marker for evaluating the survival outcome of KIRC, LGG and LUAD patients. We conducted gene enrichment analysis based on the m^7^G score, which showed higher enrichment of immune cell infiltration in the m^7^G‐low score group (Figure S9). Using CIBERSORT algorithms, we found significantly higher infiltration of resting T cells CD4 memory, resting NK cells, macrophage M1, macrophage M2, resting mast cells and neutrophils in the m^7^G‐low score group in KIRC. Similarly, the m^7^G‐low score group in LGG exhibited higher infiltration of monocytes, activated mast cells and eosinophils. In LUAD, the m^7^G‐low score group showed higher infiltration of resting T cells CD4 memory and resting mast cells (Figure 2E). Our findings suggest that m^7^G score is associated with immune cell infiltration in tumours, indicating a potential link between m^7^G writer expression and immunotherapy response in cancer patients.

## 4 Discussion

Our study comprehensively evaluated the molecular alteration and clinical relevance of m^7^G writers across the pan‐cancer cohort. We observed dysregulated expressions of m^7^G writers across various human cancers, indicating their potential significance in tumourigenesis. Intriguingly, these dysregulations were significantly associated with the prognosis of specific cancer types, suggesting that m^7^G writers may serve as prognostic marker for these cancers. Furthermore, the expression of m^7^G writers were strongly associated with the activation and inhibition of cancer‐related hallmark pathways, showing the multifaceted roles of m^7^G writers in cancer progression. Our results also revealed that the expression of m^7^G writers played a crucial role in influencing immune cell infiltration within the tumour microenvironment, particularly in cancers such as KIRC, LGG and LUAD.

In the context of our study, we emphasise the importance of m^7^G writers in human cancers as they deposit m^7^G modification on RNA, thereby mediating a variety of critical RNA metabolism processes [1]. The current understanding of m^7^G erasers and readers is in its nascent stages, making it challenging to comprehensively explore their roles and implications in human cancers [2, 7, 8]. Recent studies that included proteins involved in downstream processes relevant to m^7^G modification in their analyses need to be interpreted with caution, as there is no evidence suggesting that these ‘m^7^G‐related proteins’ directly recognise m^7^G [23, 24]. These m^7^G‐related proteins are primarily binding to the structure of the m^7^G‐cap of mRNA rather than directly recognising the m^7^G modification itself [25]. Our results clearly demonstrate that changes in m^7^G writers alone are sufficient to predict clinical outcomes across diverse cancers and the inclusion of other controversial m^7^G‐related regulators may not be necessary.

Our data underscores the significance of m^7^G writers in KIRC, the most prevalent and aggressive subtype of kidney cancer [26]. We found that aberrant expression of all m^7^G writers is associated with overall survival in KIRC patients, indicating that m^7^G writers may serve as prognostic markers and therapeutic targets in KIRC.

Immunotherapy has emerged as a promising treatment approach for KIRC [22]. However, the immunotherapy response varies among patients. Tumour‐infiltrating immune cells play a critical role in determining sensitivity of immunotherapy in KIRC. Our data demonstrate an association between the m^7^G score, developed based on the expression of m^7^G writers, and immune cell infiltration in KIRC. Higher infiltration of various types of immune cells was observed in the m^7^G score low group in KIRC. This result suggests that KIRC tumours with low m^7^G score are potentially ‘hot’ tumours associated with better response to immunotherapy [27]. We also found that low m^7^G score was positively associated with infiltration of anti‐tumourigenic M1 macrophage and pro‐tumourigenic M2 macrophage in KIRC, indicating the complexity of infiltrated immune cells within tumour and heterogeneity of tumour [28, 29]. Our findings reveal the association between m^7^G writers and immune cell infiltration within the tumour microenvironment and suggest their potential as targets for improving immunotherapy outcomes for KIRC patients.

However, there are some limitations in our study. Firstly, we only investigate the m^7^G writer expression by using the data from the TCGA database, without validation across multiple databases. The TCGA database remains the only source of multi‐omic data for more than 10 000 cancer genomes and matched normal samples across 33 cancer types [30], making it difficult to validate our pan‐cancer analysis using other resources. Second, there is a lack of experimental validation of the function of m^7^G writers in human cancer. Although our study addressed the association between the expression of m^7^G writers and human cancers, the underlying mechanisms remain unclear. Experimental studies are needed to elucidate RNAs that are affected by aberrant expression of m^7^G writers, leading to cancer development and progression.

## 5 Conclusion

In conclusion, our study indicates that m^7^G writers are dysregulated in various human cancers. These dysregulations are associated with the prognosis of specific cancers. Additionally, the expression of m^7^G writers is linked to the activation and inhibition of the cancer‐oncogenic pathways and significantly affects immune cell infiltration in the tumour microenvironment in cancers like KIRC, LGG and LUAD. Overall, our study provides a resource to infer the role of m^7^G writers in cancer and provides insights into the role of m^7^G writers as cancer biomarkers and therapeutic targets.

## Author Contributions

**Anni Su:** data curation (lead), formal analysis (lead), writing–original draft (lead), writing–review and editing (equal). **Renhua Song:** formal analysis (equal), supervision (supporting). **Justin J.‐L. Wong:** conceptualization (lead), funding acquisition (lead), project administration (lead), resources (lead), supervision (lead), writing–original draft (equal), writing–review and editing (equal).

## Ethics Statement

The authors have nothing to report.

## Conflicts of Interest

The authors declare no competing financial interest.

## Supporting information


**Figure S1.** Frequency of genetic alteration of individual m^7^G writer genes in human cancers. The bars represent the frequency of deep deletion (blue) and amplification (red) of m^7^G writer genes in each of the 33 cancer types.

**Figure S2.** Gene expression level of the individual m^7^G writer gene in human cancers. The red and blue boxes represent tumour and normal samples, respectively. The Wilcoxon test was used to compare the two groups. Significance is denoted by *p < 0.5; **p < 0.01; ***p < 0.001; ****p < 0.0001.

**Figure S3.** Gene expression level of the individual m^7^G writer gene in human cancers based on the sites of occurrence. The red and blue boxes represent tumour and normal samples, respectively. The Wilcoxon test was used to compare the two groups. Significance is denoted by *p < 0.05; **p < 0.01; ***p < 0.001; ****p < 0.0001.

**Figure S4.** Overall survival for patients with different types of cancer‐based on the expression of m^7^G writers. The forest map shows the overall survival risk ratio of six m^7^G writer genes in 33 TCGA cancer types. Significance is denoted by *p* < 0.05 by the log‐rank test.

**Figure S5.** Kaplan–Meier survival curves of KIRC patients based on the expression of m^7^G writers. Red and blue represent higher (>median) and lower (<median) levels of gene expression respectively. Significance is denoted by *p* < 0.05 log‐rank test.

**Figure S6.** Multivariate analysis of m^7^G score and main clinicopathological features in KIRC and Kaplan–Meier survival curves stratified by main clinicopathological features in KIRC. Significance is denoted by *p* < 0.05 log‐rank test.

**Figure S7.** Multivariate analysis of m^7^G score and main clinicopathological features in LGG and Kaplan–Meier survival curves stratified by main clinicopathological features in LGG. Significance is denoted by *p* < 0.05 log‐rank test.

**Figure S8.** Multivariate analysis of m^7^G score and main clinicopathological features in LUAD and Kaplan–Meier survival curves stratified by main clinicopathological features in LUAD. Significance is denoted by *p* < 0.05 log‐rank test.

**Figure S9.** Heatmap demonstrates the normalised enrichment scores of 46 immune cell infiltrates in KIRC, LGG and LUAD patients using the single‐sample Gene set Enrichment analysis (ssGESA) scores. Rows represent tumour infiltrating immune cells and columns represent samples, red and blue indicate the m^7^G‐high and ‐low groups, respectively.

**Table S1.** Frequency of mutations of m^7^G regulatory genes in 33 types of cancers.

**Table S2.** Frequency of copy number alteration of m^7^G writer genes in human cancer.

**Table S3.** The correlation between the expression of m^7^G writer genes and cancer‐related hallmark pathways.

## Data Availability

These data were derived from the following resources available in the public domain: the TCGA Research Network (https://cancergenome.nih.gov/).
