# Supplementary figures and images for: Pan‐Cancer Analysis Links Altered RNA m7G Methyltransferase Expression to Oncogenic Pathways, Immune Cell Infiltrations and Overall Survival
Source: Cancer Rep (Hoboken). 2024 Jul 23;7(7):e2138. doi: 10.1002/cnr2.2138 (PMC11264101; doi:10.1002/cnr2.2138)

Figure S1

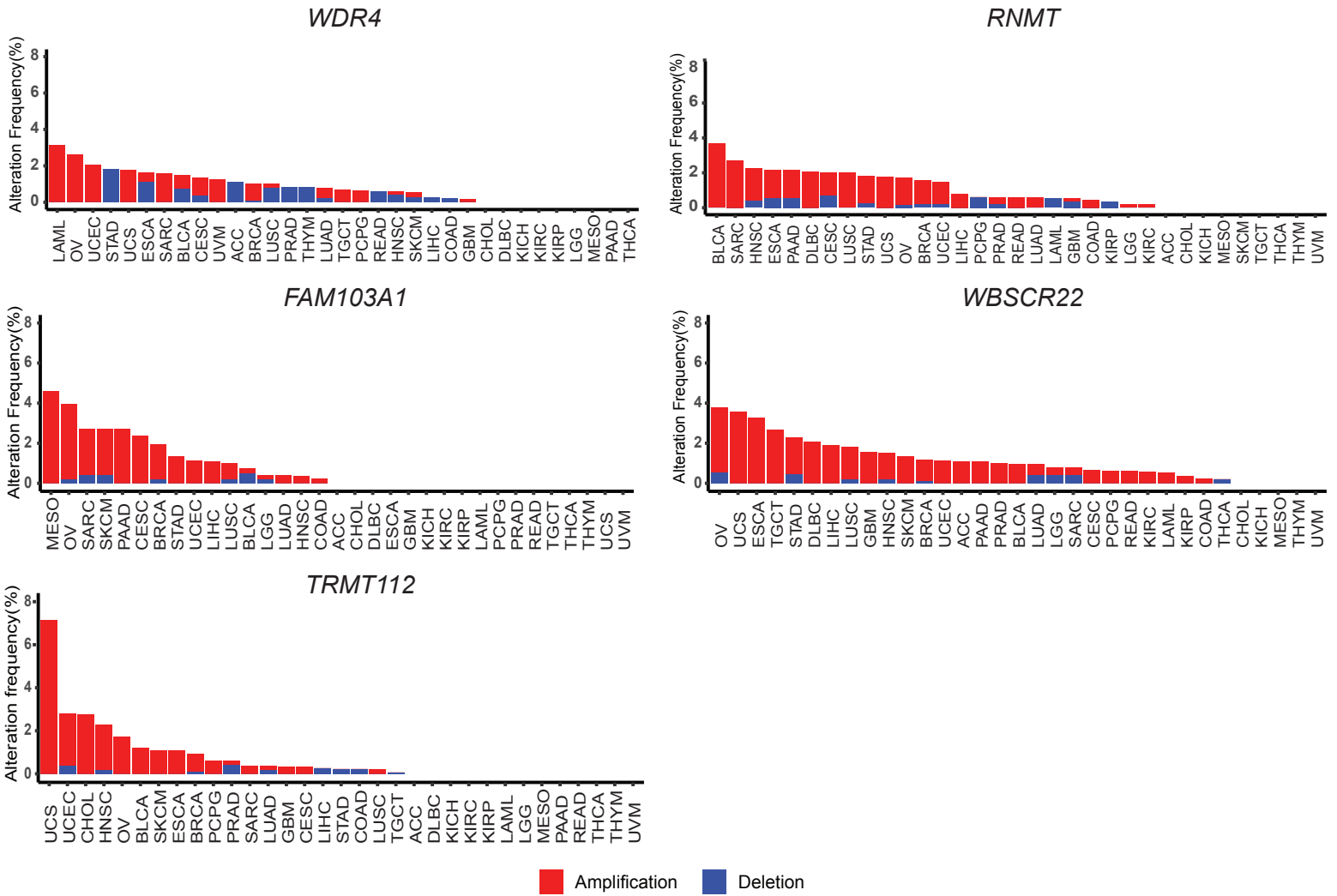

Supplement: Supplementary file 1 — Figure S1. Frequency of genetic alteration of individual m7G writer genes in human cancers. The bars represent the frequency of deep deletion (blue) and amplification (red) of m7G writer genes in each of the 33 cancer types. [file CNR2-7-e2138-s005.pdf]

*METTL1*

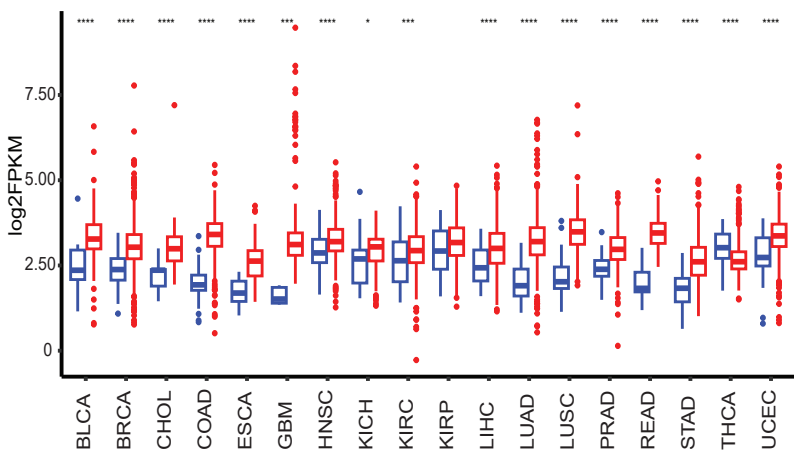

*WDR4*

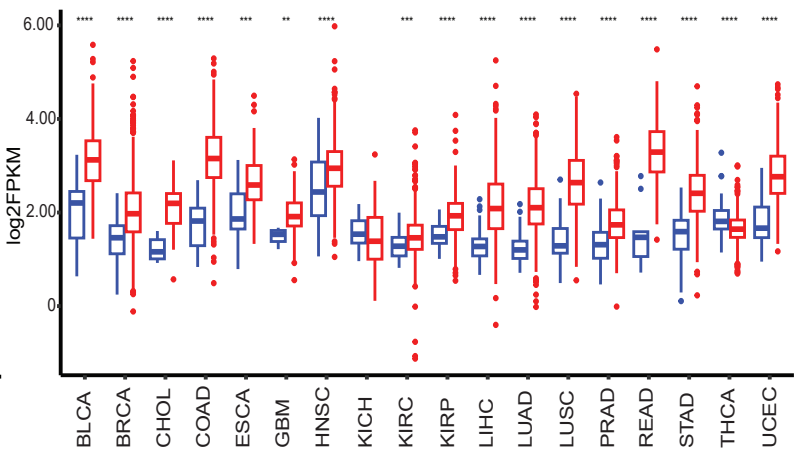

*RNMT*

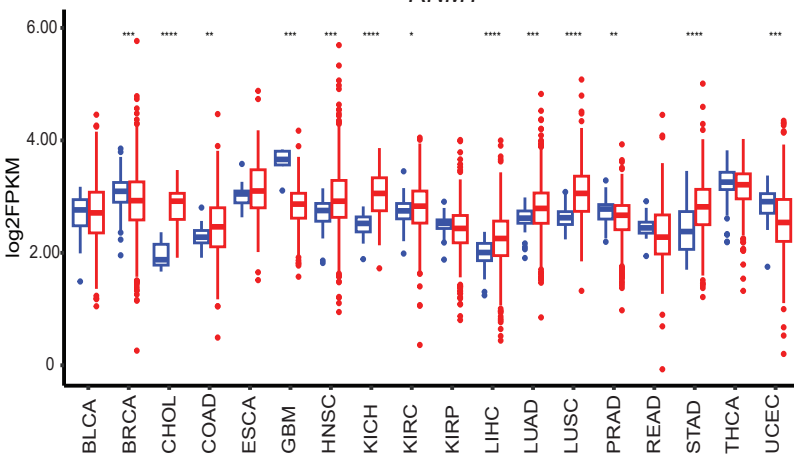

*FAM103A1*

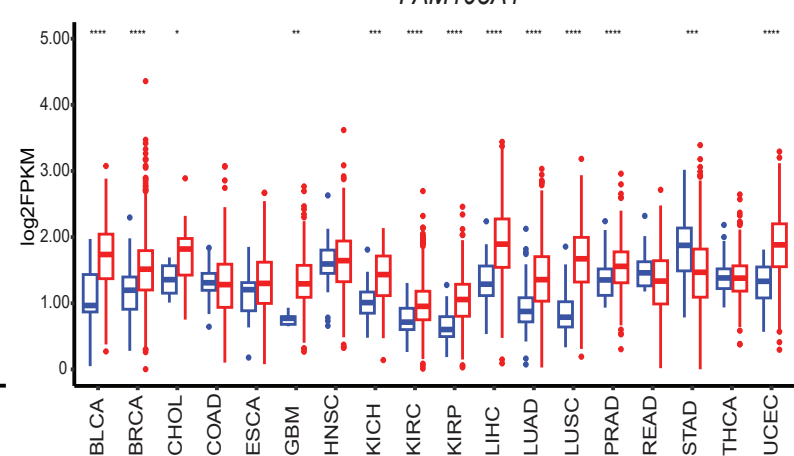

*WBSR22*

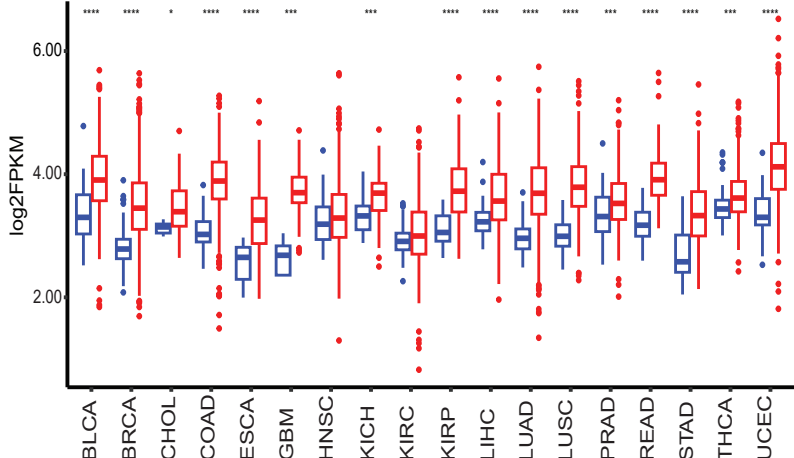

*TRMT112*

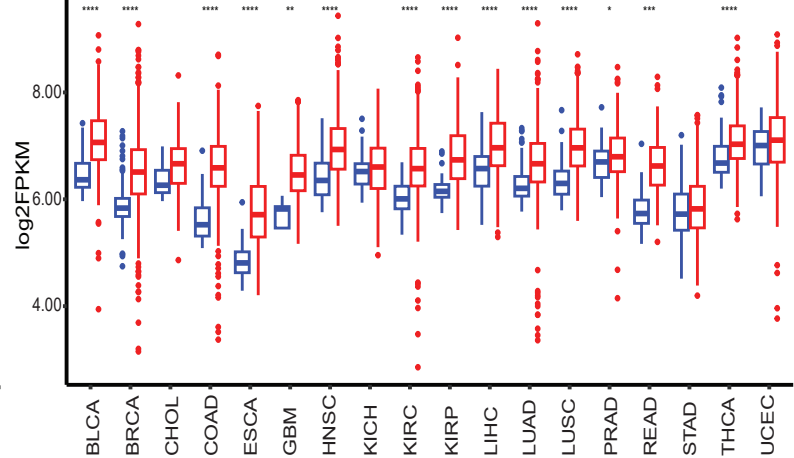

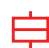 Tumour Primary 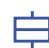 Normal Tissue

Supplement: Supplementary file 2 — Figure S2. Gene expression level of the individual m7G writer gene in human cancers. The red and blue boxes represent tumour and normal samples, respectively. The Wilcoxon test was used to compare the two groups. Significance is denoted by *p < 0.5; **p < 0.01; ***p < 0.001; ****p < 0.0001. [file CNR2-7-e2138-s008.pdf]

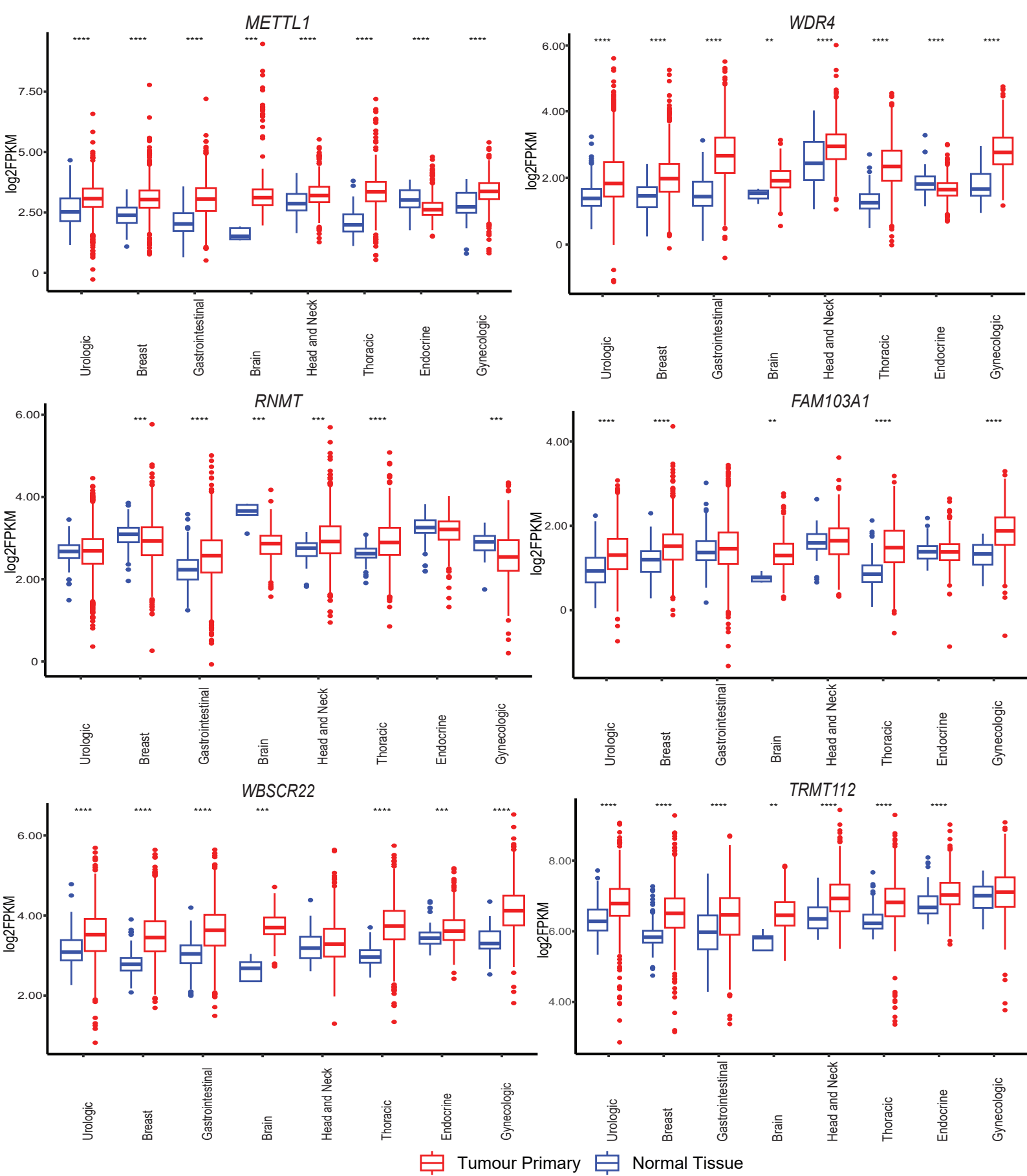

Supplement: Supplementary file 3 — Figure S3. Gene expression level of the individual m7G writer gene in human cancers based on the sites of occurrence. The red and blue boxes represent tumour and normal samples, respectively. The Wilcoxon test was used to compare the two groups. Significance is denoted by *p < 0.05; **p < 0.01; ***p < 0.001; ****p < 0.0001. [file CNR2-7-e2138-s010.pdf]

METTL1

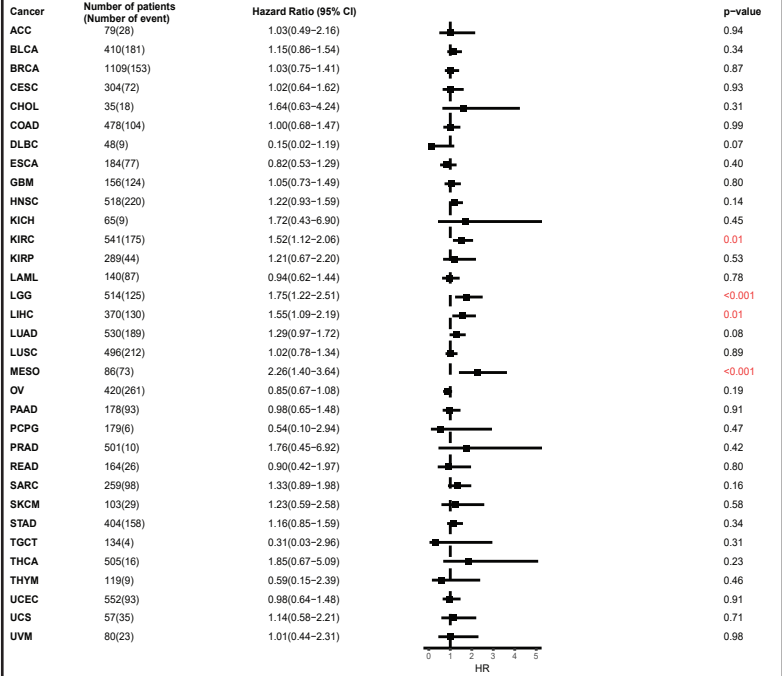

WDR4

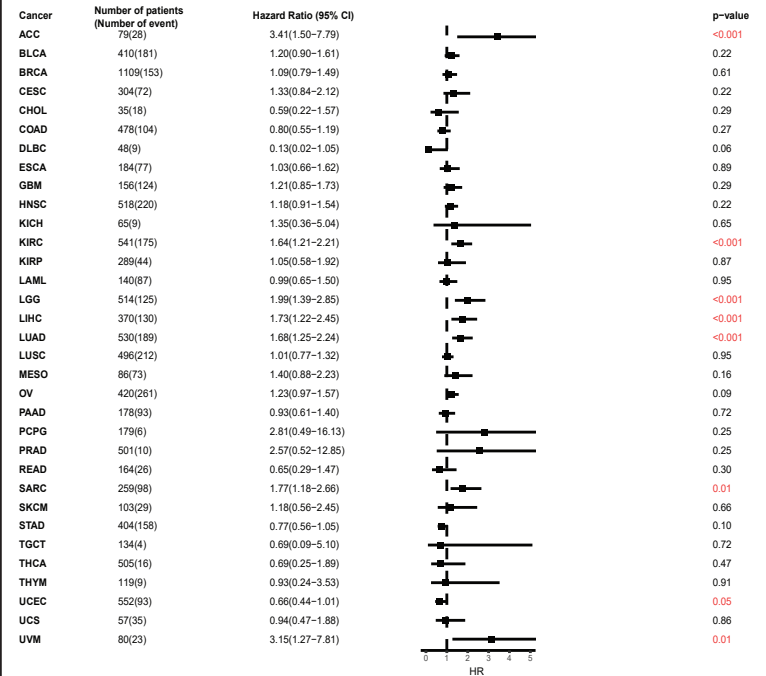

RNMT

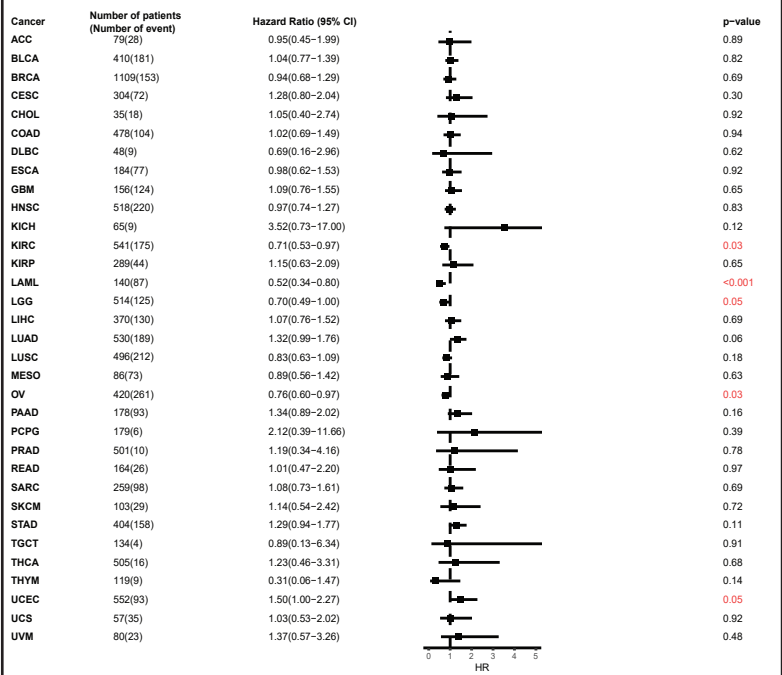

FAM103A1

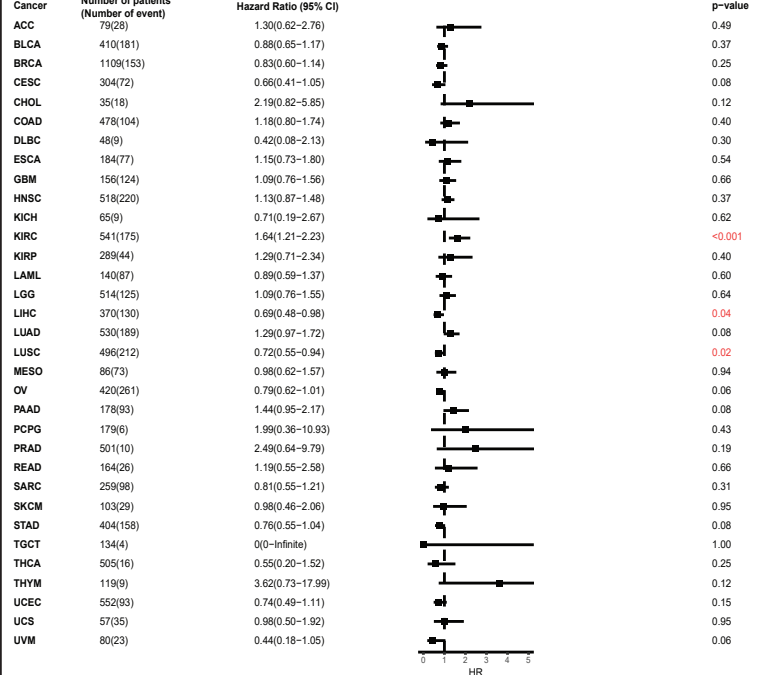

WBSCR22

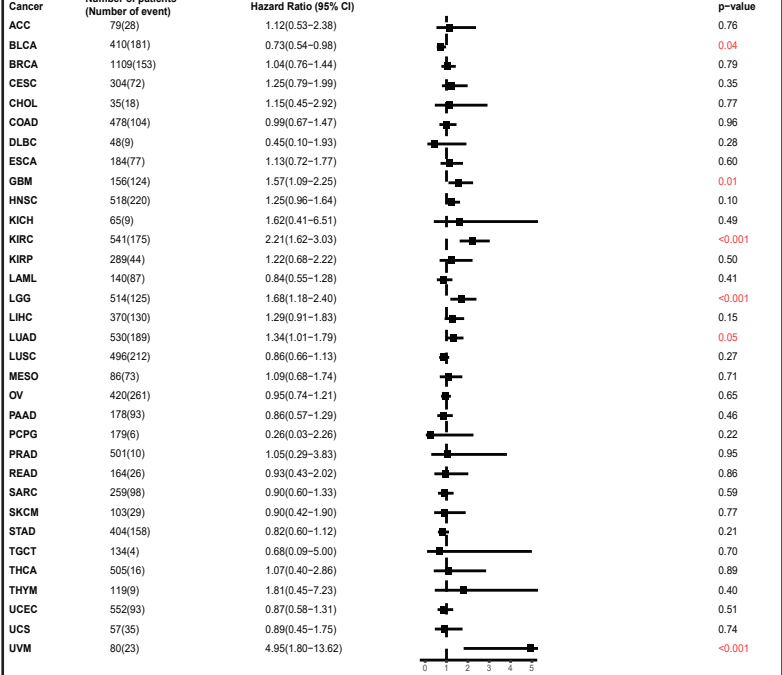

TRMT112

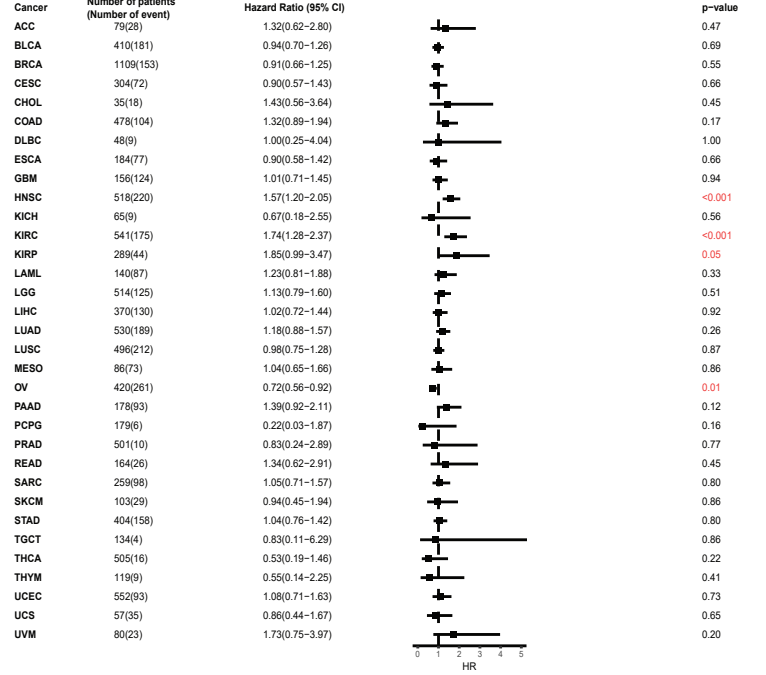

Supplement: Supplementary file 4 — Figure S4. Overall survival for patients with different types of cancer‐based on the expression of m7G writers. The forest map shows the overall survival risk ratio of six m7G writer genes in 33 TCGA cancer types. Significance is denoted by p < 0.05 by the log‐rank test. [file CNR2-7-e2138-s003.pdf]

Figure S6

KIRC

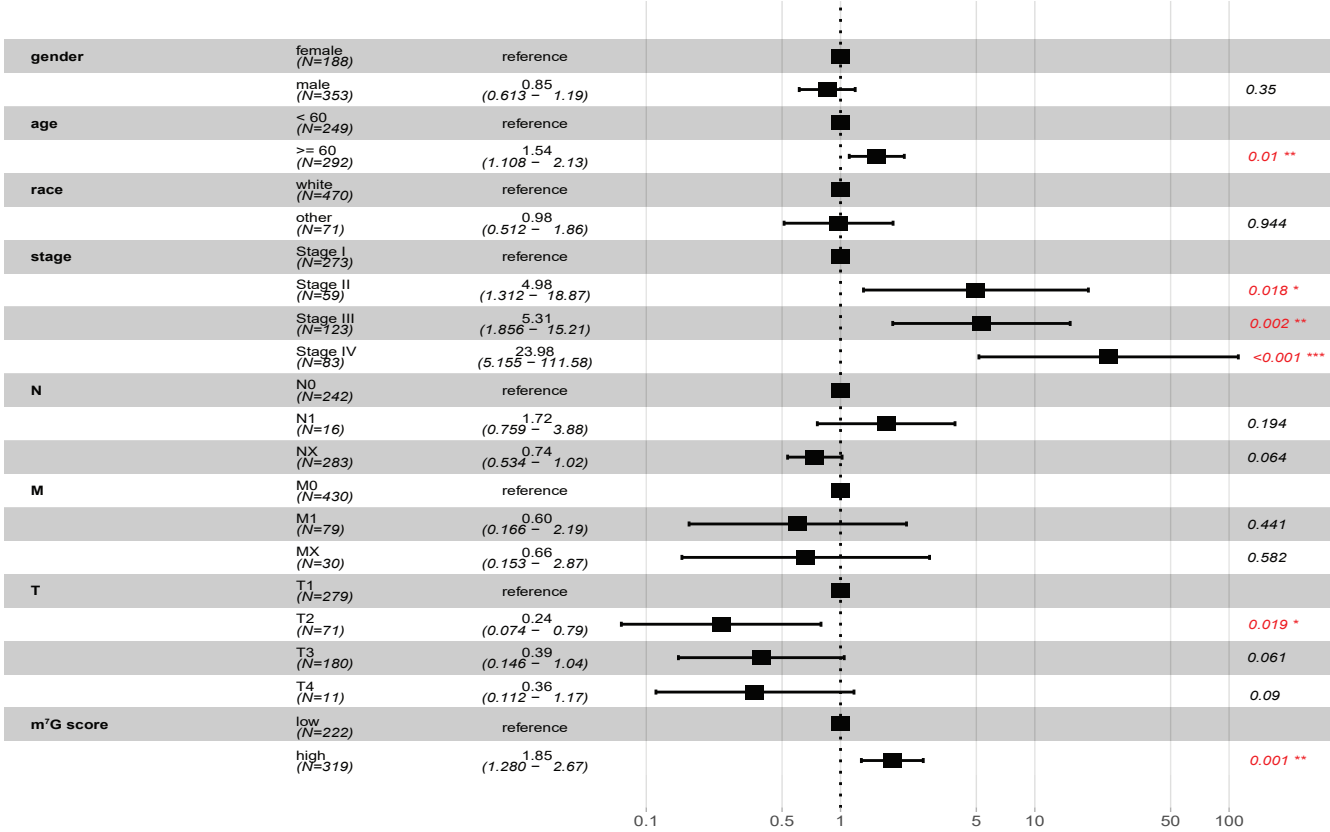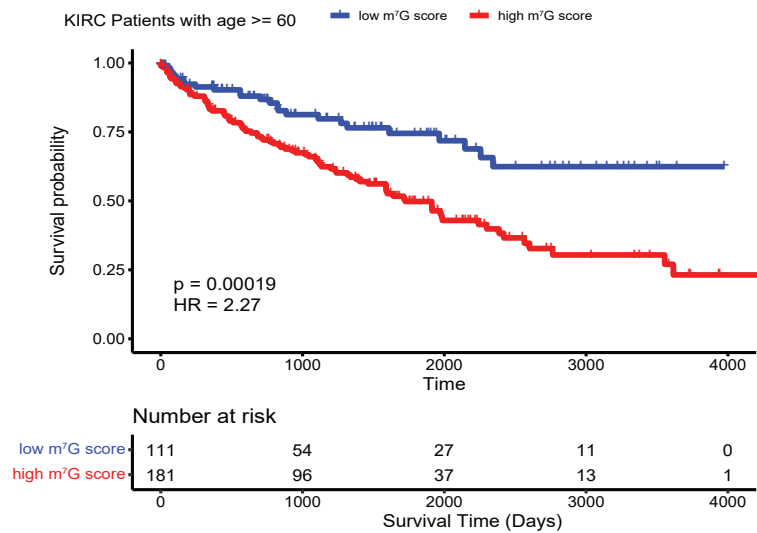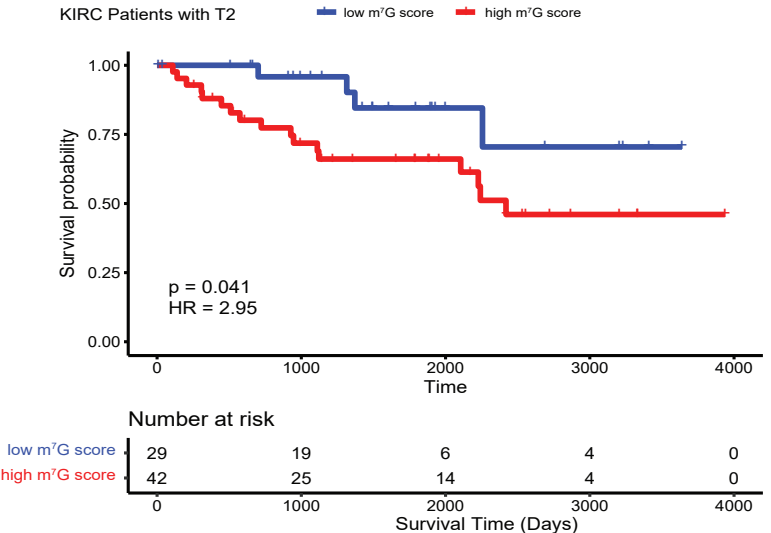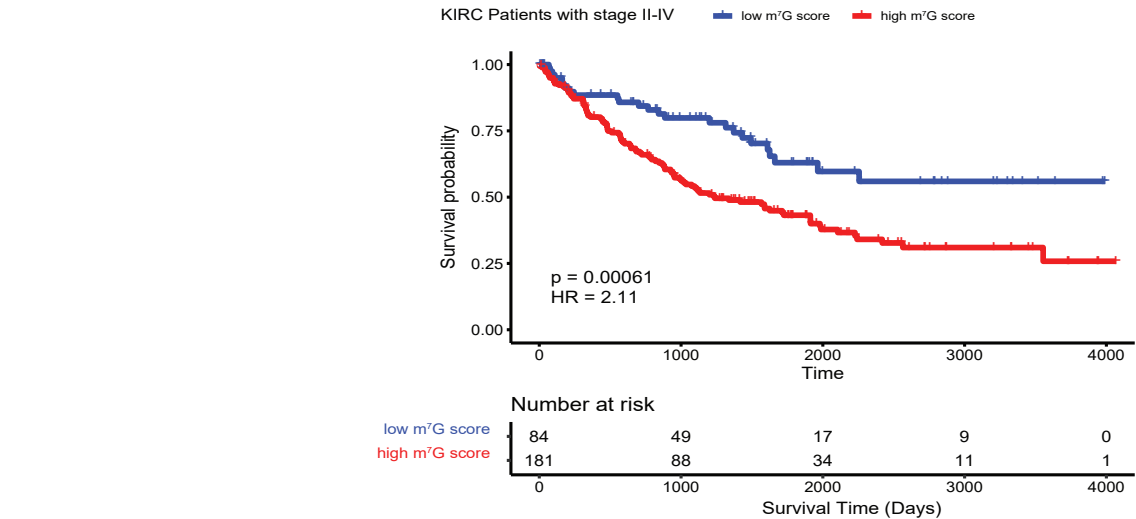

Supplement: Supplementary file 6 — Figure S6. Multivariate analysis of m7G score and main clinicopathological features in KIRC and Kaplan–Meier survival curves stratified by main clinicopathological features in KIRC. Significance is denoted by p < 0.05 log‐rank test. [file CNR2-7-e2138-s007.pdf]

Figure S7

LGG

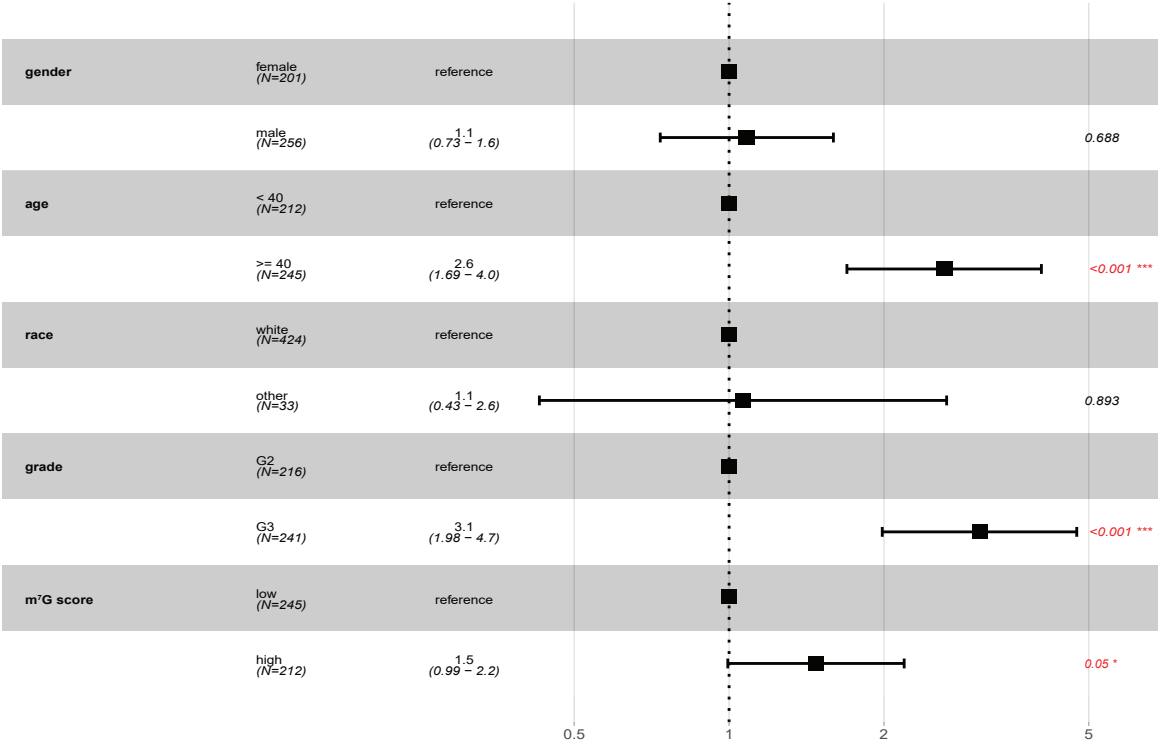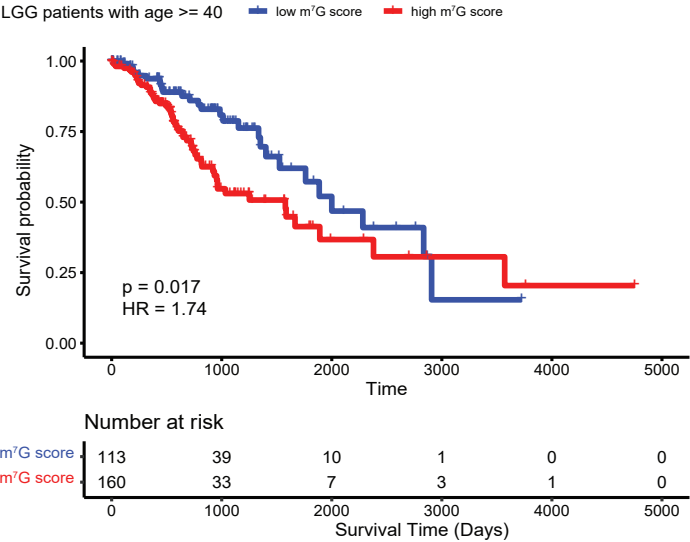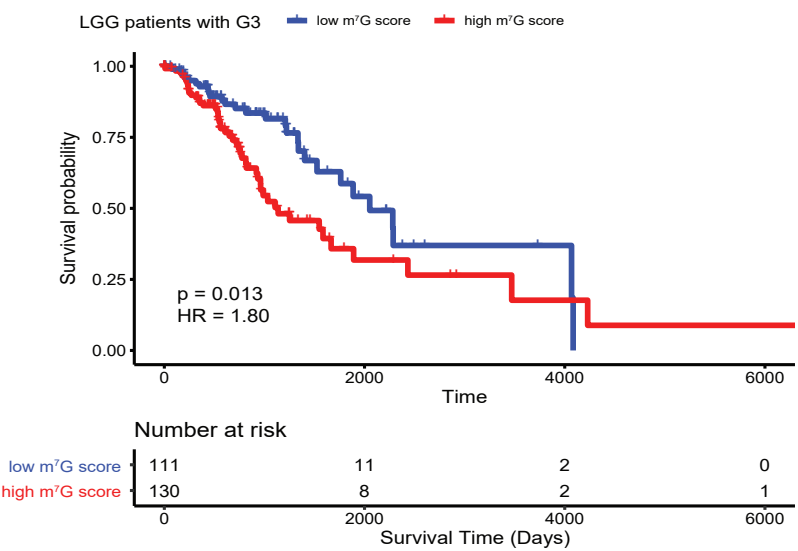

Supplement: Supplementary file 7 — Figure S7. Multivariate analysis of m7G score and main clinicopathological features in LGG and Kaplan–Meier survival curves stratified by main clinicopathological features in LGG. Significance is denoted by p < 0.05 log‐rank test. [file CNR2-7-e2138-s004.pdf]

Figure S8

LUAD

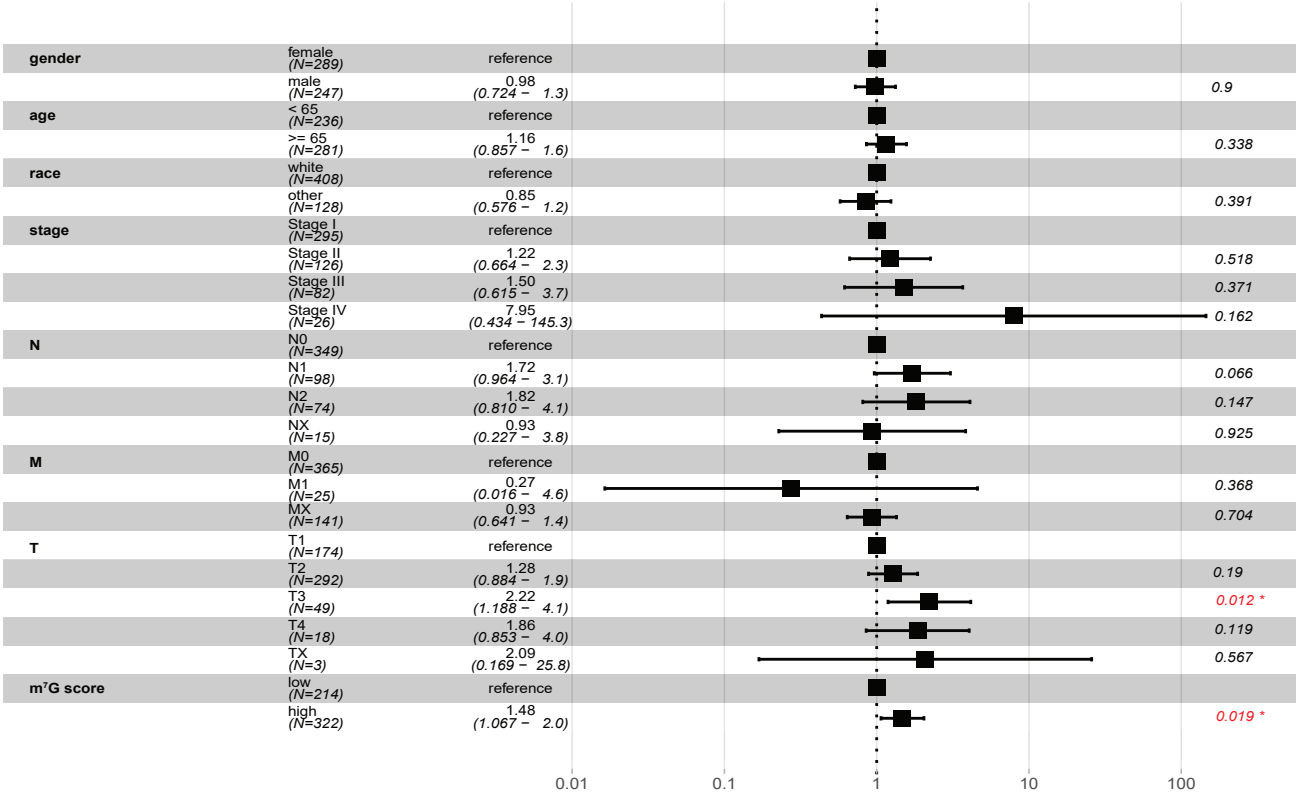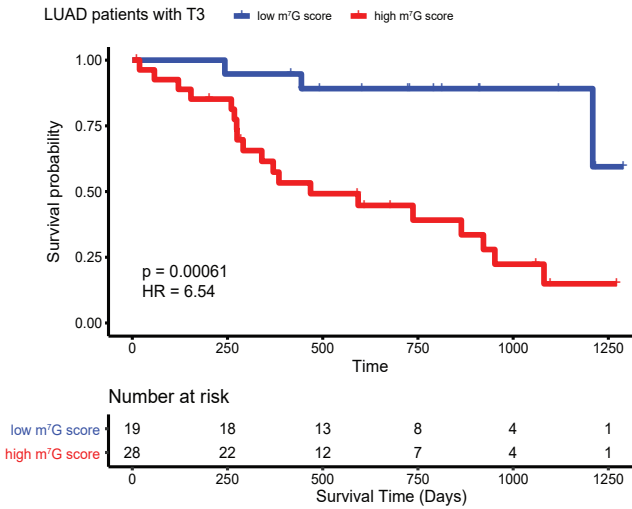

Supplement: Supplementary file 8 — Figure S8. Multivariate analysis of m7G score and main clinicopathological features in LUAD and Kaplan–Meier survival curves stratified by main clinicopathological features in LUAD. Significance is denoted by p < 0.05 log‐rank test. [file CNR2-7-e2138-s001.pdf]

Figure S9

KIRC

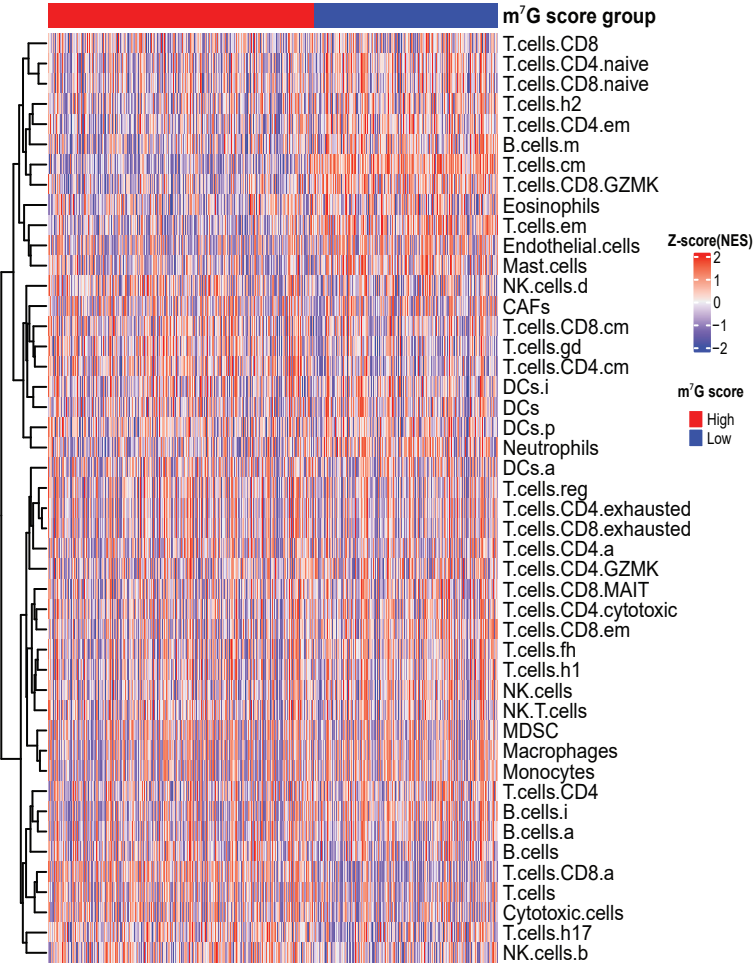

LGG

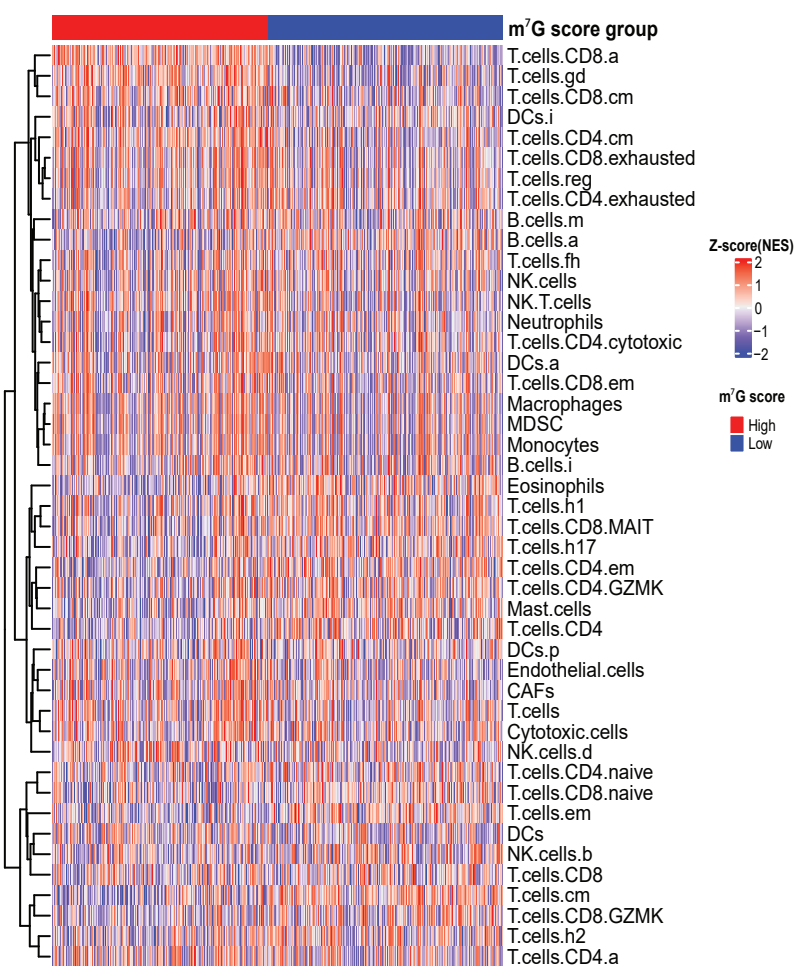

LUAD

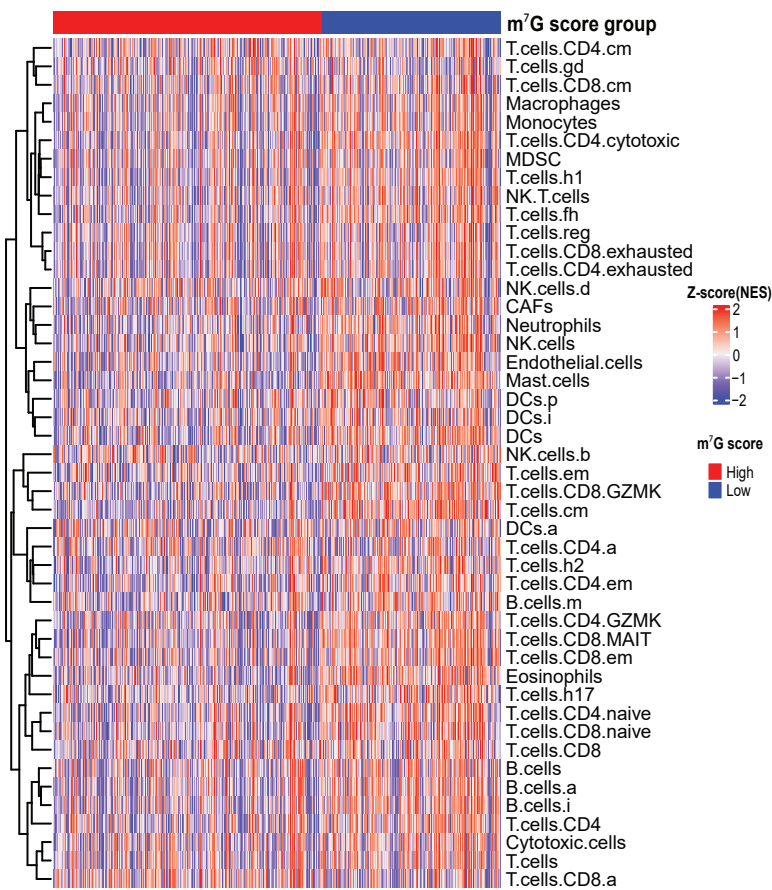

Supplement: Supplementary file 9 — Figure S9. Heatmap demonstrates the normalised enrichment scores of 46 immune cell infiltrates in KIRC, LGG and LUAD patients using the single‐sample Gene set Enrichment analysis (ssGESA) scores. Rows represent tumour infiltrating immune cells and columns represent samples, red and blue indicate the m7G‐high and ‐low groups, respectively. [file CNR2-7-e2138-s006.pdf]
