# Supplementary material for: Pan‐Cancer Analysis Links Altered RNA m7G Methyltransferase Expression to Oncogenic Pathways, Immune Cell Infiltrations and Overall Survival
Source: Cancer Rep (Hoboken). 2024 Jul 23;7(7):e2138. doi: 10.1002/cnr2.2138 (PMC11264101; doi:10.1002/cnr2.2138)
Supplement: Supplementary file 5 — Figure S5. Kaplan–Meier survival curves of KIRC patients based on the expression of m7G writers. Red and blue represent higher (>median) and lower (<median) levels of gene expression respectively. Significance is denoted by p < 0.05 log‐rank test. [file CNR2-7-e2138-s011.pdf]

Figure S5

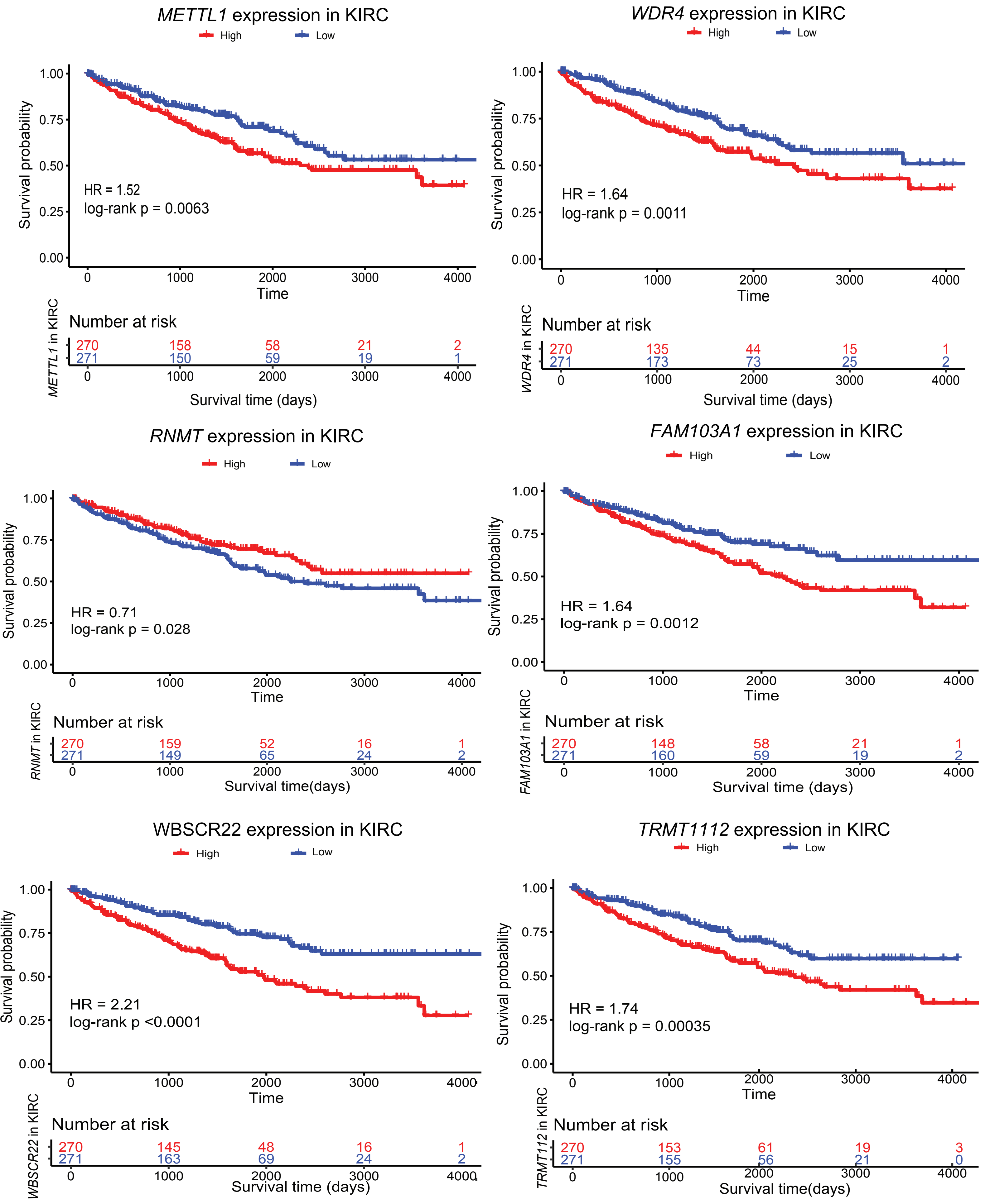

METTL1 expression in KIRC
High
Low
Survival probability
1.00
0.75
0.50
0.25
0.00
HR = 1.52
log-rank p = 0.0063
0
1000
2000
3000
4000
Time
METTL1 in KIRC
Number at risk
270 158 58 21 2
271 150 59 19 1
0 1000 2000 3000 4000
Survival time (days)
WDR4 expression in KIRC
High
Low
Survival probability
1.00
0.75
0.50
0.25
0.00
HR = 1.64
log-rank p = 0.0011
0
1000
2000
3000
4000
Time
WDR4 in KIRC
Number at risk
270 135 44 15 1
271 173 73 25 2
0 1000 2000 3000 4000
Survival time (days)
RNMT expression in KIRC
High
Low
Survival probability
1.00
0.75
0.50
0.25
0.00
HR = 0.71
log-rank p = 0.028
0
1000
2000
3000
4000
Time
RNMT in KIRC
Number at risk
270 159 52 16 1
271 149 65 24 2
0 1000 2000 3000 4000
Survival time(days)
FAM103A1 expression in KIRC
High
Low
Survival probability
1.00
0.75
0.50
0.25
0.00
HR = 1.64
log-rank p = 0.0012
0
1000
2000
3000
4000
Time
FAM103A1 in KIRC
Number at risk
270 148 58 21 1
271 160 59 19 2
0 1000 2000 3000 4000
Survival time (days)
WBSCR22 expression in KIRC
High
Low
Survival probability
1.00
0.75
0.50
0.25
0.00
HR = 2.21
log-rank p <0.0001
0
1000
2000
3000
4000
Time
WBSCR22 in KIRC
Number at risk
270 145 48 16 1
271 163 69 24 2
0 1000 2000 3000 4000
Survival time (days)
TRMT1112 expression in KIRC
High
Low
Survival probability
1.00
0.75
0.50
0.25
0.00
HR = 1.74
log-rank p = 0.00035
0
1000
2000
3000
4000
Time
TRMT112 in KIRC
Number at risk
270 153 61 19 3
271 155 56 21 0
0 1000 2000 3000 4000
Survival time(days)
