# Supplementary material for: Pan‐Cancer Analysis Links Altered RNA m7G Methyltransferase Expression to Oncogenic Pathways, Immune Cell Infiltrations and Overall Survival
Source: Cancer Rep (Hoboken). 2024 Jul 23;7(7):e2138. doi: 10.1002/cnr2.2138 (PMC11264101; doi:10.1002/cnr2.2138)
Supplement: Supplementary file 10 — Table S1. Frequency of mutations of m7G regulatory genes in 33 types of cancers. [file CNR2-7-e2138-s002.pdf]

Table S1

Gene	BRCA	ACC	BLCA	CESC	CHOL	COAD	DLBC	ESCA	GBM	HNSC	KIRC
METTL1	0.100705	0	0	0.515464	0	0.649351	0	0	0.344828	1.433692	0
WDR4	0.60423	1.111111	1.538462	1.030928	0	2.597403	2.083333	1.081081	0.344828	0.358423	0
RNMT	0.100705	0	0	1.030928	0	1.948052	0	1.081081	0	0.716846	0.228833
FAM103A1	0.100705	0	0	0	0	0	0	0	0	0	0
WBSCR22	0.302115	1.111111	2.307692	0	2.857143	0.649351	0	0	0	0	0
TRMT112	0.503525	0	0.769231	0	0	0.649351	0	0	0	0.358423	0
**Total sample size**	993	90	130	194	35	154	48	185	290	279	437

Gene	KIRP	LAML	LGG	LIHC	KICH	LUAD	LUSC	MESO	OV	PAAD	PCPG
METTL1	0.621118	0	0	1.515152	0	0	0	0	0	0	0.543478
WDR4	0	0	0	0	0	0.434783	1.685393	1.204819	0.632911	0	0
RNMT	1.242236	0	0	0	1.515152	2.608696	2.247191	0	0	0	0
FAM103A1	0	0	0	0	0	0	0	0	0	0	0
WBSCR22	0	0	0	0.505051	0	0.434783	1.685393	0	0.316456	0	0
TRMT112	0.621118	0	0	0	0	0	0	0	0	0	0
**Total sample size**	161	197	286	198	66	230	178	83	316	150	184

Gene	PRAD	READ	SARC	SKCM	STAD	TGCT	THCA	THYM	UCEC	UCS	UVM	% across 33 types of human cancer
METTL1	0	0	2.024291	0.869565	1.038062	0	0	0	2.419355	0	0	0.414708322
WDR4	0.301205	1.449275	0	0.869565	1.038062	0.645161	0	0	1.612903	0	0	0.552944429
RNMT	0.301205	1.449275	0.404858	2.898551	3.460208	0	0	0	2.822581	1.754386	0	0.76029859
FAM103A1	0	0	0	0	0	0	0	0	2.016129	0	0	0.082941664
WBSCR22	0.301205	1.449275	0.404858	0.57971	2.076125	0.645161	0	0	2.419355	0	0	0.456179154
TRMT112	0	0	0	0.289855	0.346021	0	0	0	0.403226	0	0	0.165883329
**Total sample size**	332	69	247	345	289	155	405	123	248	57	80	7234
