# Supplementary material for: Pan‐Cancer Analysis Links Altered RNA m7G Methyltransferase Expression to Oncogenic Pathways, Immune Cell Infiltrations and Overall Survival
Source: Cancer Rep (Hoboken). 2024 Jul 23;7(7):e2138. doi: 10.1002/cnr2.2138 (PMC11264101; doi:10.1002/cnr2.2138)
Supplement: Supplementary file 11 — Table S2. Frequency of copy number alteration of m7G writer genes in human cancer. [file CNR2-7-e2138-s012.pdf]

Table S2

Gene	Type of genetic alteration	ACC	BLCA	BRCA	CESC	CHOL	COAD	DLBC	ESCA	GBM	HNSC	KICH	KIRC	KIRP	LAML	LGG	LIHC	LUAD	LUSC	MESO	OV	PAAD	PCPG	PRAD	READ	SARC	SKCM	STAD	TGCT	THCA	THYM	UCEC	UCS	UVM
METTL1	Amplification	6.666667	1.470588	1.574074	0.677966	2.777778	0	2.083333	0.543478	13.69151	0.766284	0	0.189394	0	0	4.093567	1.891892	5.813953	0.598802	0	1.899827	2.173913	1.234568	0.406504	0.606061	17.50973	3.542234	3.174603	0	0	0	0.371058	0	0
	Deletion	0	0	0	0	0	0	0	0	0	0	0	0	0	0	0.389864	0	0	0	0	0.172712	0	0	0.203252	0	0	0	0	0	0	0	0	0	0
WDR4	Amplification	0	0.735294	0.925926	1.016949	0	0	0	0.543478	0.17331	0.191571	0	0	0	3.141361	0	0	0.581395	0.199601	0	2.590674	0	0.617284	0	0	1.55642	0.27248	0	0.666667	0	0	2.040816	1.785714	1.25
	Deletion	1.111111	0.735294	0.092593	0.338983	0	0.221729	0	1.086957	0	0.383142	0	0	0	0	0	0.27027	0.193798	0.798403	0	0	0	0	0.813008	0.606061	0	0.27248	1.814059	0	0	0.813008	0	0	0
RNMT	Amplification	0	3.676471	1.388889	1.355932	0	0.443459	2.083333	1.630435	0.17331	1.915709	0	0.189394	0	0	0.194932	0.810811	0.581395	1.996008	0	1.554404	1.630435	0	0.406504	0.606061	2.723735	0	1.587302	0	0	0	1.298701	1.785714	0
	Deletion	0	0	0.185185	0.677966	0	0	0	0.543478	0.34662	0.383142	0	0	0.347222	0.52356	0	0	0	0	0	0.172712	0.543478	0.617284	0.203252	0	0	0	0.226757	0	0	0	0.185529	0	0
FAM103A1	Amplification	0	0.245098	1.759259	2.372881	0	0.221729	0	0	0	0.383142	0	0	0	0	0.194932	1.081081	0.387597	0.798403	4.597701	3.799655	2.717391	0	0	0	2.33463	2.33463	1.360544	0	0	0	1.113173	0	0
	Deletion	0	0.490196	0.185185	0	0	0	0	0	0	0	0	0	0	0	0.194932	0	0	0.199601	0	0.172712	0	0	0	0	0.389105	0.389105	0	0	0	0	0	0	0
WBSCR22	Amplification	1.111111	0.980392	1.111111	0.677966	0	0.221729	2.083333	3.26087	1.559792	1.340996	0	0.568182	0.347222	0.52356	0.389864	1.891892	0.581395	1.596806	0	3.28152	1.086957	0.617284	1.01626	0.606061	0.389105	1.362398	1.814059	2.666667	0	0	1.113173	3.571429	0
	Deletion	0	0	0.092593	0	0	0	0	0	0	0.191571	0	0	0	0	0.389864	0	0.387597	0.199601	0	0.518135	0	0	0	0	0.389105	0	0.453515	0	0.200401	0	0	0	0
TRMT112	Amplification	0	1.22549	0.833333	0.338983	2.777778	0	0	1.086957	0.34662	2.10728	0	0	0	0	0	0	0.193798	0.199601	0	1.727116	0	0.617284	0.203252	0	0.389105	1.089918	0	0	0	0	2.411874	7.142857	0
	Deletion	0	0	0.092593	0	0	0.221729	0	0	0	0.191571	0	0	0	0	0	0.27027	0.193798	0	0	0	0	0	0.406504	0	0	0	0.226757	0.066667	0	0	0.371058	0	0
