# Supplementary material for: Pan‐Cancer Analysis Links Altered RNA m7G Methyltransferase Expression to Oncogenic Pathways, Immune Cell Infiltrations and Overall Survival
Source: Cancer Rep (Hoboken). 2024 Jul 23;7(7):e2138. doi: 10.1002/cnr2.2138 (PMC11264101; doi:10.1002/cnr2.2138)
Supplement: Supplementary file 12 — Table S3. The correlation between the expression of m7G writer genes and cancer‐related hallmark pathways. [file CNR2-7-e2138-s009.pdf]

Table S3

Pathways	Genes	Cancers	PCC	pvalue	padj	Correlations	IDs
HALLMARK_ADIPOGENESIS	METTL1	DLBC	0.5947567	8.28E-06	4.06E-04	positive	P1
HALLMARK_ADIPOGENESIS	WDR4	DLBC	0.5105945	2.09E-04	8.99E-03	positive	P1
HALLMARK_ADIPOGENESIS	WBSCR22	DLBC	0.5459184	5.99E-05	2.39E-03	positive	P1
HALLMARK_ADIPOGENESIS	TRMT112	GBM	0.5340159	5.92E-13	2.60E-11	positive	P1
HALLMARK_ANDROGEN_RESPONSE	WBSCR22	KIRP	-0.5914893	0	0	negative	N2
HALLMARK_ANDROGEN_RESPONSE	TRMT112	SKCM	-0.5656057	4.81E-10	2.41E-08	negative	N2
HALLMARK_ANDROGEN_RESPONSE	WBSCR22	THCA	-0.6013532	0	0	negative	N2
HALLMARK_ANDROGEN_RESPONSE	TRMT112	THCA	-0.5427883	0	0	negative	N2
HALLMARK_ANDROGEN_RESPONSE	WBSCR22	THYM	-0.6887164	0.00E+00	0.00E+00	negative	N2
HALLMARK_ANDROGEN_RESPONSE	RNMT	THYM	0.6771823	0.00E+00	0.00E+00	positive	P2
HALLMARK_ANDROGEN_RESPONSE	TRMT112	THYM	-0.6861872	0.00E+00	0.00E+00	negative	N2
HALLMARK_ANDROGEN_RESPONSE	RNMT	UVM	0.6051199	2.76E-09	1.27E-07	positive	P2
HALLMARK_ANDROGEN_RESPONSE	TRMT112	UVM	-0.6656214	1.63E-11	7.81E-10	negative	N2
HALLMARK_ANGIOGENESIS	METTL1	TGCT	-0.5263631	4.58E-12	2.06E-10	negative	N3
HALLMARK_ANGIOGENESIS	WBSCR22	TGCT	-0.6822736	0.00E+00	0.00E+00	negative	N3
HALLMARK_ANGIOGENESIS	FAM103A1	TGCT	-0.6737644	0.00E+00	0.00E+00	negative	N3
HALLMARK_ANGIOGENESIS	FAM103A1	UVM	-0.5530062	1.04E-07	5.10E-06	negative	N3
HALLMARK_APICAL_JUNCTION	METTL1	TGCT	-0.5386601	1.16E-12	5.45E-11	negative	N4
HALLMARK_APICAL_JUNCTION	WBSCR22	TGCT	-0.6473068	0.00E+00	0.00E+00	negative	N4
HALLMARK_APICAL_JUNCTION	FAM103A1	TGCT	-0.6965908	0.00E+00	0.00E+00	negative	N4
HALLMARK_APICAL_JUNCTION	WBSCR22	THYM	-0.5052372	3.98E-09	1.47E-07	negative	N4
HALLMARK_APICAL_JUNCTION	RNMT	UVM	-0.5678738	3.94E-08	1.73E-06	negative	N4
HALLMARK_APICAL_JUNCTION	FAM103A1	UVM	-0.5264772	5.27E-07	2.37E-05	negative	N4
HALLMARK_APICAL_SURFACE	WBSCR22	TGCT	-0.5009962	6.60E-11	2.51E-09	negative	N5
HALLMARK_APICAL_SURFACE	FAM103A1	TGCT	-0.6324703	0.00E+00	0.00E+00	negative	N5
HALLMARK_APICAL_SURFACE	RNMT	UVM	-0.5634305	5.29E-08	2.22E-06	negative	N5
HALLMARK_APICAL_SURFACE	FAM103A1	UVM	-0.5284948	4.68E-07	2.15E-05	negative	N5
HALLMARK_APOPTOSIS	RNMT	LAML	-0.5230217	5.60E-12	2.58E-10	negative	N6
HALLMARK_BILE_ACID_METABOLISM	METTL1	KICH	-0.5153471	9.52E-06	4.47E-04	negative	N7
HALLMARK_BILE_ACID_METABOLISM	TRMT112	KICH	-0.5292793	4.90E-06	2.35E-04	negative	N7
HALLMARK_BILE_ACID_METABOLISM	FAM103A1	TGCT	-0.5457674	5.10E-13	1.84E-11	negative	N7
HALLMARK_CHOLESTEROL_HOMEOSTASIS	FAM103A1	TGCT	-0.5739553	1.62E-14	6.48E-13	negative	N8
HALLMARK_CHOLESTEROL_HOMEOSTASIS	FAM103A1	UCS	-0.5004761	7.35E-05	0.003677187	negative	N8
HALLMARK_CHOLESTEROL_HOMEOSTASIS	RNMT	UVM	-0.5072832	1.57E-06	5.96E-05	negative	N8
HALLMARK_CHOLESTEROL_HOMEOSTASIS	TRMT112	UVM	0.5473933	1.49E-07	6.68E-06	positive	P8
HALLMARK_COAGULATION	FAM103A1	TGCT	-0.5485417	3.69E-13	1.36E-11	negative	N9
HALLMARK_COAGULATION	RNMT	UVM	-0.5019887	2.10E-06	7.75E-05	negative	N9
HALLMARK_COMPLEMENT	METTL1	LAML	-0.5226016	5.87E-12	2.70E-10	negative	N10
HALLMARK_COMPLEMENT	RNMT	LAML	-0.5149664	1.33E-11	5.87E-10	negative	N10
HALLMARK_DNA_REPAIR	TRMT112	BLCA	0.5114859	0.00E+00	0.00E+00	positive	P11
HALLMARK_DNA_REPAIR	METTL1	BRCA	0.5329774	0	0	positive	P11
HALLMARK_DNA_REPAIR	TRMT112	BRCA	0.5950161	0	0	positive	P11
HALLMARK_DNA_REPAIR	WBSCR22	BRCA	0.5633315	0	0	positive	P11
HALLMARK_DNA_REPAIR	METTL1	CESC	0.5183722	0	0	positive	P11
HALLMARK_DNA_REPAIR	TRMT112	CESC	0.5461829	0	0	positive	P11
HALLMARK_DNA_REPAIR	WBSCR22	CHOL	0.6716458	9.99E-06	0.000499724	positive	P11
HALLMARK_DNA_REPAIR	METTL1	COAD	0.6348424	0	0	positive	P11
HALLMARK_DNA_REPAIR	TRMT112	COAD	0.6360449	0	0	positive	P11
HALLMARK_DNA_REPAIR	METTL1	DLBC	0.5156638	1.76E-04	7.93E-03	positive	P11
HALLMARK_DNA_REPAIR	WDR4	DLBC	0.728468	4.39E-09	2.11E-07	positive	P11
HALLMARK_DNA_REPAIR	TRMT112	DLBC	0.6563699	4.08E-07	1.92E-05	positive	P11
HALLMARK_DNA_REPAIR	WBSCR22	DLBC	0.7197773	8.15E-09	3.83E-07	positive	P11
HALLMARK_DNA_REPAIR	METTL1	ESCA	0.5453615	1.33E-15	6.66E-14	positive	P11
HALLMARK_DNA_REPAIR	TRMT112	ESCA	0.635963	0.00E+00	0.00E+00	positive	P11
HALLMARK_DNA_REPAIR	TRMT112	GBM	0.6442237	0.00E+00	0.00E+00	positive	P11
HALLMARK_DNA_REPAIR	WBSCR22	GBM	0.5006645	2.43E-11	1.22E-09	positive	P11
HALLMARK_DNA_REPAIR	METTL1	HNSC	0.5199824	0	0	positive	P11
HALLMARK_DNA_REPAIR	TRMT112	HNSC	0.587036	0	0	positive	P11
HALLMARK_DNA_REPAIR	METTL1	KICH	0.5464673	2.07E-06	9.93E-05	positive	P11
HALLMARK_DNA_REPAIR	TRMT112	KICH	0.5426605	2.51E-06	1.23E-04	positive	P11
HALLMARK_DNA_REPAIR	WBSCR22	KICH	0.5005355	1.87E-05	8.79E-04	positive	P11
HALLMARK_DNA_REPAIR	METTL1	KIRC	0.5779771	0	0	positive	P11
HALLMARK_DNA_REPAIR	TRMT112	KIRC	0.6888329	0	0	positive	P11
HALLMARK_DNA_REPAIR	FAM103A1	KIRC	0.5313736	0	0	positive	P11
HALLMARK_DNA_REPAIR	WBSCR22	KIRC	0.5677098	0	0	positive	P11

HALLMARK_DNA_REPAIR	METTL1	KIRP	0.5521944	0	0	positive	P11
HALLMARK_DNA_REPAIR	TRMT112	KIRP	0.6560098	0	0	positive	P11
HALLMARK_DNA_REPAIR	TRMT112	LAML	0.5275607	3.40E-12	1.70E-10	positive	P11
HALLMARK_DNA_REPAIR	WBSCR22	LGG	0.6752353	0	0	positive	P11
HALLMARK_DNA_REPAIR	TRMT112	LGG	0.6578815	0	0	positive	P11
HALLMARK_DNA_REPAIR	METTL1	LIHC	0.570882	0	0	positive	P11
HALLMARK_DNA_REPAIR	WBSCR22	LIHC	0.5079269	0	0	positive	P11
HALLMARK_DNA_REPAIR	TRMT112	LIHC	0.6386023	0	0	positive	P11
HALLMARK_DNA_REPAIR	TRMT112	LUAD	0.6414638	0	0	positive	P11
HALLMARK_DNA_REPAIR	TRMT112	LUSC	0.6023942	0	0	positive	P11
HALLMARK_DNA_REPAIR	WDR4	MESO	0.556837	2.13E-08	1.05E-06	positive	P11
HALLMARK_DNA_REPAIR	TRMT112	MESO	0.5172458	2.90E-07	1.36E-05	positive	P11
HALLMARK_DNA_REPAIR	METTL1	OV	0.5358205	0	0	positive	P11
HALLMARK_DNA_REPAIR	TRMT112	OV	0.6370827	0	0	positive	P11
HALLMARK_DNA_REPAIR	WBSCR22	PAAD	0.6201054	0.00E+00	0.00E+00	positive	P11
HALLMARK_DNA_REPAIR	TRMT112	PAAD	0.6226029	0	0	positive	P11
HALLMARK_DNA_REPAIR	METTL1	PRAD	0.6907528	0	0	positive	P11
HALLMARK_DNA_REPAIR	WBSCR22	PRAD	0.6962223	0	0	positive	P11
HALLMARK_DNA_REPAIR	RNMT	PRAD	-0.5333367	0	0	negative	N11
HALLMARK_DNA_REPAIR	TRMT112	PRAD	0.689751	0	0	positive	P11
HALLMARK_DNA_REPAIR	METTL1	READ	0.5938867	0.00E+00	0.00E+00	positive	P11
HALLMARK_DNA_REPAIR	WBSCR22	SARC	0.5007719	0	0	positive	P11
HALLMARK_DNA_REPAIR	WDR4	SARC	0.519691	0	0	positive	P11
HALLMARK_DNA_REPAIR	TRMT112	SARC	0.6002269	0	0	positive	P11
HALLMARK_DNA_REPAIR	TRMT112	SKCM	0.5306675	8.13E-09	3.90E-07	positive	P11
HALLMARK_DNA_REPAIR	TRMT112	STAD	0.5042596	0	0	positive	P11
HALLMARK_DNA_REPAIR	WDR4	TGCT	0.602389	4.44E-16	1.95E-14	positive	P11
HALLMARK_DNA_REPAIR	TRMT112	TGCT	0.5550553	1.70E-13	8.31E-12	positive	P11
HALLMARK_DNA_REPAIR	WBSCR22	THCA	0.5462289	0	0	positive	P11
HALLMARK_DNA_REPAIR	TRMT112	THCA	0.6679386	0	0	positive	P11
HALLMARK_DNA_REPAIR	WBSCR22	THYM	0.6455662	1.78E-15	7.64E-14	positive	P11
HALLMARK_DNA_REPAIR	WDR4	THYM	0.6865308	0.00E+00	0.00E+00	positive	P11
HALLMARK_DNA_REPAIR	TRMT112	THYM	0.6500854	8.88E-16	4.09E-14	positive	P11
HALLMARK_DNA_REPAIR	TRMT112	UCEC	0.5991327	0	0	positive	P11
HALLMARK_DNA_REPAIR	TRMT112	UCS	0.5587424	6.27E-06	2.95E-04	positive	P11
HALLMARK_DNA_REPAIR	METTL1	UVM	0.6782959	4.76E-12	2.33E-10	positive	P11
HALLMARK_DNA_REPAIR	RNMT	UVM	-0.6642326	1.86E-11	8.91E-10	negative	N11
HALLMARK_DNA_REPAIR	TRMT112	UVM	0.6844564	2.56E-12	1.28E-10	positive	P11
HALLMARK_E2F_TARGETS	WDR4	DLBC	0.5491261	5.31E-05	2.39E-03	positive	P12
HALLMARK_E2F_TARGETS	WDR4	TGCT	0.6869812	0.00E+00	0.00E+00	positive	P12
HALLMARK_EPITHELIAL_MESENCHYMAL_TRANSITION	METTL1	TGCT	-0.5066751	3.70E-11	1.63E-09	negative	N13
HALLMARK_EPITHELIAL_MESENCHYMAL_TRANSITION	WBSCR22	TGCT	-0.6480146	0.00E+00	0.00E+00	negative	N13
HALLMARK_EPITHELIAL_MESENCHYMAL_TRANSITION	FAM103A1	TGCT	-0.6861707	0.00E+00	0.00E+00	negative	N13
HALLMARK_ESTROGEN_RESPONSE_EARLY	TRMT112	ACC	-0.5194675	9.32E-07	4.57E-05	negative	N14
HALLMARK_ESTROGEN_RESPONSE_EARLY	WBSCR22	LGG	-0.5657137	0	0	negative	N14
HALLMARK_ESTROGEN_RESPONSE_EARLY	METTL1	TGCT	-0.5554138	1.63E-13	7.80E-12	negative	N14
HALLMARK_ESTROGEN_RESPONSE_EARLY	WBSCR22	TGCT	-0.5857474	3.55E-15	1.53E-13	negative	N14
HALLMARK_ESTROGEN_RESPONSE_EARLY	FAM103A1	TGCT	-0.7059058	0.00E+00	0.00E+00	negative	N14
HALLMARK_ESTROGEN_RESPONSE_EARLY	WBSCR22	THCA	-0.5259754	0	0	negative	N14
HALLMARK_ESTROGEN_RESPONSE_EARLY	TRMT112	UCS	-0.5643519	4.83E-06	2.32E-04	negative	N14
HALLMARK_ESTROGEN_RESPONSE_LATE	FAM103A1	TGCT	-0.5518578	2.49E-13	9.45E-12	negative	N15
HALLMARK_ESTROGEN_RESPONSE_LATE	FAM103A1	UVM	-0.5397996	2.38E-07	1.12E-05	negative	N15
HALLMARK_FATTY_ACID_METABOLISM	METTL1	DLBC	0.5607698	3.39E-05	1.59E-03	positive	P16
HALLMARK_FATTY_ACID_METABOLISM	TRMT112	GBM	0.5729818	4.44E-15	2.04E-13	positive	P16
HALLMARK_FATTY_ACID_METABOLISM	WBSCR22	THYM	0.511106	2.45E-09	9.31E-08	positive	P16
HALLMARK_G2M_CHECKPOINT	RNMT	SKCM	0.5259024	1.17E-08	5.71E-07	positive	P17
HALLMARK_G2M_CHECKPOINT	WDR4	TGCT	0.6562764	0.00E+00	0.00E+00	positive	P17
HALLMARK_G2M_CHECKPOINT	WBSCR22	THCA	-0.5262076	0	0	negative	N17
HALLMARK_GLYCOLYSIS	WDR4	UVM	0.6011321	3.73E-09	1.79E-07	positive	P18
HALLMARK_GLYCOLYSIS	FAM103A1	UVM	-0.5444451	1.79E-07	8.57E-06	negative	N18
HALLMARK_HEDGEHOG_SIGNALING	RNMT	CHOL	0.5516519	0.000592311	0.02961556	positive	P19
HALLMARK_HEDGEHOG_SIGNALING	WBSCR22	DLBC	-0.6043504	5.40E-06	2.38E-04	negative	N19
HALLMARK_HEDGEHOG_SIGNALING	TRMT112	GBM	-0.6586124	0.00E+00	0.00E+00	negative	N19
HALLMARK_HEDGEHOG_SIGNALING	FAM103A1	GBM	-0.5328322	6.80E-13	3.27E-11	negative	N19
HALLMARK_HEDGEHOG_SIGNALING	WBSCR22	KIRP	-0.5288861	0	0	negative	N19
HALLMARK_HEDGEHOG_SIGNALING	TRMT112	OV	-0.5251242	0	0	negative	N19
HALLMARK_HEDGEHOG_SIGNALING	TRMT112	PCPG	-0.6165892	0.00E+00	0.00E+00	negative	N19

HALLMARK_HEDGEHOG_SIGNALING	METTL1	PRAD	-0.565941	0	0	negative	N19
HALLMARK_HEDGEHOG_SIGNALING	WBSCR22	PRAD	-0.5939308	0	0	negative	N19
HALLMARK_HEDGEHOG_SIGNALING	TRMT112	PRAD	-0.554867	0	0	negative	N19
HALLMARK_HEDGEHOG_SIGNALING	FAM103A1	SKCM	-0.5610087	7.11E-10	3.56E-08	negative	N19
HALLMARK_HEDGEHOG_SIGNALING	METTL1	TGCT	-0.6496037	0.00E+00	0.00E+00	negative	N19
HALLMARK_HEDGEHOG_SIGNALING	WBSCR22	TGCT	-0.6319844	0.00E+00	0.00E+00	negative	N19
HALLMARK_HEDGEHOG_SIGNALING	FAM103A1	TGCT	-0.6863544	0.00E+00	0.00E+00	negative	N19
HALLMARK_HEDGEHOG_SIGNALING	WBSCR22	THCA	-0.505813	0	0	negative	N19
HALLMARK_HEDGEHOG_SIGNALING	WBSCR22	THYM	-0.7515188	0.00E+00	0.00E+00	negative	N19
HALLMARK_HEDGEHOG_SIGNALING	RNMT	THYM	0.5456393	1.16E-10	5.10E-09	positive	P19
HALLMARK_HEDGEHOG_SIGNALING	TRMT112	THYM	-0.5737925	7.36E-12	3.24E-10	negative	N19
HALLMARK_HEDGEHOG_SIGNALING	TRMT112	UCS	-0.5061704	5.90E-05	2.65E-03	negative	N19
HALLMARK_HEME_METABOLISM	WDR4	DLBC	-0.5626564	3.15E-05	1.45E-03	negative	N20
HALLMARK_HEME_METABOLISM	TRMT112	DLBC	-0.5688068	2.46E-05	1.13E-03	negative	N20
HALLMARK_HEME_METABOLISM	WBSCR22	DLBC	-0.5503679	5.06E-05	2.08E-03	negative	N20
HALLMARK_HEME_METABOLISM	METTL1	KICH	-0.6182291	3.18E-08	1.59E-06	negative	N20
HALLMARK_HEME_METABOLISM	TRMT112	KICH	-0.6572763	2.04E-09	1.02E-07	negative	N20
HALLMARK_HEME_METABOLISM	FAM103A1	KICH	-0.5084843	1.31E-05	6.40E-04	negative	N20
HALLMARK_HEME_METABOLISM	WBSCR22	KICH	-0.5719595	5.26E-07	2.63E-05	negative	N20
HALLMARK_HEME_METABOLISM	WBSCR22	KIRP	-0.5396887	0	0	negative	N20
HALLMARK_HEME_METABOLISM	WBSCR22	LGG	-0.5468654	0	0	negative	N20
HALLMARK_HEME_METABOLISM	TRMT112	PCPG	-0.5512675	1.33E-15	6.26E-14	negative	N20
HALLMARK_HEME_METABOLISM	WBSCR22	TGCT	-0.5287069	3.54E-12	1.42E-10	negative	N20
HALLMARK_HEME_METABOLISM	WBSCR22	THCA	-0.5334974	0	0	negative	N20
HALLMARK_HEME_METABOLISM	TRMT112	THCA	-0.5901833	0	0	negative	N20
HALLMARK_HEME_METABOLISM	WBSCR22	THYM	-0.6794256	0.00E+00	0.00E+00	negative	N20
HALLMARK_HEME_METABOLISM	RNMT	THYM	0.6196958	4.49E-14	2.15E-12	positive	P20
HALLMARK_HEME_METABOLISM	WDR4	THYM	-0.5478748	9.40E-11	4.04E-09	negative	N20
HALLMARK_HEME_METABOLISM	TRMT112	THYM	-0.7149678	0.00E+00	0.00E+00	negative	N20
HALLMARK_HEME_METABOLISM	TRMT112	UCS	-0.5657972	4.51E-06	2.21E-04	negative	N20
HALLMARK_HYPOXIA	FAM103A1	TGCT	-0.5861882	3.11E-15	1.27E-13	negative	N21
HALLMARK_IL6_JAK_STAT3_SIGNALING	TRMT112	DLBC	-0.5056982	2.46E-04	1.06E-02	negative	N22
HALLMARK_IL6_JAK_STAT3_SIGNALING	WBSCR22	DLBC	-0.5687976	2.46E-05	1.03E-03	negative	N22
HALLMARK_IL6_JAK_STAT3_SIGNALING	METTL1	LAML	-0.5065757	3.22E-11	1.45E-09	negative	N22
HALLMARK_IL6_JAK_STAT3_SIGNALING	RNMT	LAML	-0.5233066	5.43E-12	2.55E-10	negative	N22
HALLMARK_IL6_JAK_STAT3_SIGNALING	WBSCR22	PAAD	-0.5500535	1.78E-15	8.35E-14	negative	N22
HALLMARK_INFLAMMATORY_RESPONSE	WBSCR22	DLBC	-0.5212313	1.46E-04	5.65E-03	negative	N23
HALLMARK_INFLAMMATORY_RESPONSE	WBSCR22	PAAD	-0.527812	3.73E-14	1.72E-12	negative	N23
HALLMARK_INTERFERON_GAMMA_RESPONSE	METTL1	LAML	-0.502251	5.01E-11	2.21E-09	negative	N24
HALLMARK_KRAS_SIGNALING_DN	WBSCR22	CHOL	-0.5652185	4.03E-04	0.019362563	negative	N25
HALLMARK_KRAS_SIGNALING_DN	TRMT112	DLBC	-0.5194367	1.55E-04	6.81E-03	negative	N25
HALLMARK_KRAS_SIGNALING_DN	TRMT112	GBM	-0.5259121	1.52E-12	6.52E-11	negative	N25
HALLMARK_KRAS_SIGNALING_DN	WBSCR22	LGG	-0.5057882	0	0	negative	N25
HALLMARK_KRAS_SIGNALING_DN	RNMT	UVM	-0.6632811	2.03E-11	9.54E-10	negative	N25
HALLMARK_KRAS_SIGNALING_UP	WBSCR22	DLBC	-0.5213473	1.45E-04	5.65E-03	negative	N26
HALLMARK_KRAS_SIGNALING_UP	METTL1	LAML	-0.5530733	1.79E-13	8.39E-12	negative	N26
HALLMARK_KRAS_SIGNALING_UP	TRMT112	MESO	-0.5011508	7.65E-07	3.52E-05	negative	N26
HALLMARK_KRAS_SIGNALING_UP	WBSCR22	PAAD	-0.5622765	4.44E-16	2.13E-14	negative	N26
HALLMARK_KRAS_SIGNALING_UP	TRMT112	THYM	-0.5509143	7.05E-11	3.03E-09	negative	N26
HALLMARK_MITOTIC_SPINDLE	WBSCR22	BLCA	-0.5004887	0.00E+00	0.00E+00	negative	N27
HALLMARK_MITOTIC_SPINDLE	WBSCR22	CESC	-0.5410099	0	0	negative	N27
HALLMARK_MITOTIC_SPINDLE	RNMT	DLBC	0.6484403	6.24E-07	3.12E-05	positive	P27
HALLMARK_MITOTIC_SPINDLE	TRMT112	DLBC	-0.6653214	2.48E-07	1.22E-05	negative	N27
HALLMARK_MITOTIC_SPINDLE	WBSCR22	DLBC	-0.7385682	2.07E-09	1.01E-07	negative	N27
HALLMARK_MITOTIC_SPINDLE	RNMT	GBM	0.5133536	6.20E-12	3.10E-10	positive	P27
HALLMARK_MITOTIC_SPINDLE	TRMT112	GBM	-0.6610138	0.00E+00	0.00E+00	negative	N27
HALLMARK_MITOTIC_SPINDLE	FAM103A1	GBM	-0.5625516	1.75E-14	8.77E-13	negative	N27
HALLMARK_MITOTIC_SPINDLE	WBSCR22	HNSC	-0.5034892	0	0	negative	N27
HALLMARK_MITOTIC_SPINDLE	FAM103A1	KICH	-0.5855698	2.41E-07	1.21E-05	negative	N27
HALLMARK_MITOTIC_SPINDLE	WBSCR22	KICH	-0.527667	5.30E-06	2.60E-04	negative	N27
HALLMARK_MITOTIC_SPINDLE	TRMT112	KIRC	-0.5599911	0	0	negative	N27
HALLMARK_MITOTIC_SPINDLE	RNMT	KIRP	0.5503833	0	0	positive	P27
HALLMARK_MITOTIC_SPINDLE	WBSCR22	KIRP	-0.578396	0	0	negative	N27
HALLMARK_MITOTIC_SPINDLE	RNMT	LIHC	0.5590371	0	0	positive	P27
HALLMARK_MITOTIC_SPINDLE	TRMT112	OV	-0.5191955	0	0	negative	N27
HALLMARK_MITOTIC_SPINDLE	WBSCR22	PCPG	-0.5709059	0	0	negative	N27
HALLMARK_MITOTIC_SPINDLE	TRMT112	PCPG	-0.6117577	0.00E+00	0.00E+00	negative	N27

HALLMARK_MITOTIC_SPINDLE	METTL1	PRAD	-0.5306208	0	0	negative	N27
HALLMARK_MITOTIC_SPINDLE	WBSCR22	PRAD	-0.6245454	0	0	negative	N27
HALLMARK_MITOTIC_SPINDLE	RNMT	PRAD	0.5651192	0	0	positive	P27
HALLMARK_MITOTIC_SPINDLE	TRMT112	PRAD	-0.611285	0	0	negative	N27
HALLMARK_MITOTIC_SPINDLE	METTL1	THCA	-0.5227962	0	0	negative	N27
HALLMARK_MITOTIC_SPINDLE	WBSCR22	THCA	-0.7309264	0	0	negative	N27
HALLMARK_MITOTIC_SPINDLE	TRMT112	THCA	-0.593618	0	0	negative	N27
HALLMARK_MITOTIC_SPINDLE	WBSCR22	THYM	-0.5574801	3.75E-11	1.46E-09	negative	N27
HALLMARK_MITOTIC_SPINDLE	RNMT	THYM	0.5581132	3.53E-11	1.62E-09	positive	P27
HALLMARK_MITOTIC_SPINDLE	TRMT112	THYM	-0.6142701	8.57E-14	3.86E-12	negative	N27
HALLMARK_MITOTIC_SPINDLE	WBSCR22	UCEC	-0.5021231	0	0	negative	N27
HALLMARK_MITOTIC_SPINDLE	RNMT	UCEC	0.5499287	0	0	positive	P27
HALLMARK_MITOTIC_SPINDLE	TRMT112	UVM	-0.6041631	2.97E-09	1.39E-07	negative	N27
HALLMARK_MTORC1_SIGNALING	WDR4	DLBC	0.5019335	2.78E-04	1.17E-02	positive	P28
HALLMARK_MTORC1_SIGNALING	WDR4	TGCT	0.6687609	0.00E+00	0.00E+00	positive	P28
HALLMARK_MTORC1_SIGNALING	WDR4	UVM	0.5394653	2.43E-07	1.12E-05	positive	P28
HALLMARK_MYC_TARGETS_V1	WDR4	ACC	0.5453078	2.03E-07	1.02E-05	positive	P29
HALLMARK_MYC_TARGETS_V1	METTL1	CESC	0.5274735	0	0	positive	P29
HALLMARK_MYC_TARGETS_V1	WBSCR22	CHOL	0.6459454	2.78E-05	0.001361331	positive	P29
HALLMARK_MYC_TARGETS_V1	WDR4	DLBC	0.75289	6.72E-10	3.29E-08	positive	P29
HALLMARK_MYC_TARGETS_V1	TRMT112	ESCA	0.5522457	4.44E-16	2.18E-14	positive	P29
HALLMARK_MYC_TARGETS_V1	WDR4	HNSC	0.5311132	0	0	positive	P29
HALLMARK_MYC_TARGETS_V1	METTL1	LAML	0.5712719	1.87E-14	9.14E-13	positive	P29
HALLMARK_MYC_TARGETS_V1	METTL1	LIHC	0.5836014	0	0	positive	P29
HALLMARK_MYC_TARGETS_V1	WDR4	LIHC	0.5419122	0	0	positive	P29
HALLMARK_MYC_TARGETS_V1	TRMT112	OV	0.5581618	0	0	positive	P29
HALLMARK_MYC_TARGETS_V1	WDR4	SARC	0.5778663	0	0	positive	P29
HALLMARK_MYC_TARGETS_V1	WDR4	TGCT	0.7208953	0.00E+00	0.00E+00	positive	P29
HALLMARK_MYC_TARGETS_V1	WDR4	THYM	0.61938	4.66E-14	2.24E-12	positive	P29
HALLMARK_MYC_TARGETS_V2	WDR4	ACC	0.5135947	1.29E-06	6.35E-05	positive	P30
HALLMARK_MYC_TARGETS_V2	METTL1	BLCA	0.5007874	0.00E+00	0.00E+00	positive	P30
HALLMARK_MYC_TARGETS_V2	METTL1	BRCA	0.5644631	0	0	positive	P30
HALLMARK_MYC_TARGETS_V2	METTL1	CESC	0.6414686	0	0	positive	P30
HALLMARK_MYC_TARGETS_V2	METTL1	CHOL	0.5489953	0.000637372	0.03186859	positive	P30
HALLMARK_MYC_TARGETS_V2	WBSCR22	CHOL	0.5357552	9.10E-04	0.042788112	positive	P30
HALLMARK_MYC_TARGETS_V2	METTL1	COAD	0.6167176	0	0	positive	P30
HALLMARK_MYC_TARGETS_V2	WDR4	COAD	0.5751281	0	0	positive	P30
HALLMARK_MYC_TARGETS_V2	TRMT112	COAD	0.521113	0	0	positive	P30
HALLMARK_MYC_TARGETS_V2	METTL1	DLBC	0.5460897	5.95E-05	2.74E-03	positive	P30
HALLMARK_MYC_TARGETS_V2	WDR4	DLBC	0.7599921	3.74E-10	1.87E-08	positive	P30
HALLMARK_MYC_TARGETS_V2	METTL1	ESCA	0.5400414	2.66E-15	1.31E-13	positive	P30
HALLMARK_MYC_TARGETS_V2	WDR4	ESCA	0.5034911	3.22E-13	1.61E-11	positive	P30
HALLMARK_MYC_TARGETS_V2	WDR4	GBM	0.5135491	6.07E-12	3.04E-10	positive	P30
HALLMARK_MYC_TARGETS_V2	METTL1	HNSC	0.5611338	0	0	positive	P30
HALLMARK_MYC_TARGETS_V2	WDR4	HNSC	0.6071731	0	0	positive	P30
HALLMARK_MYC_TARGETS_V2	METTL1	KICH	0.5661045	7.28E-07	3.56E-05	positive	P30
HALLMARK_MYC_TARGETS_V2	METTL1	KIRC	0.6750288	0	0	positive	P30
HALLMARK_MYC_TARGETS_V2	WBSCR22	KIRC	0.5070397	0	0	positive	P30
HALLMARK_MYC_TARGETS_V2	METTL1	KIRP	0.5914723	0	0	positive	P30
HALLMARK_MYC_TARGETS_V2	METTL1	LAML	0.7678562	0.00E+00	0.00E+00	positive	P30
HALLMARK_MYC_TARGETS_V2	WDR4	LAML	0.57256	1.58E-14	7.88E-13	positive	P30
HALLMARK_MYC_TARGETS_V2	WBSCR22	LGG	0.5698423	0	0	positive	P30
HALLMARK_MYC_TARGETS_V2	WDR4	LGG	0.5301371	0	0	positive	P30
HALLMARK_MYC_TARGETS_V2	METTL1	LIHC	0.6659245	0	0	positive	P30
HALLMARK_MYC_TARGETS_V2	WBSCR22	LIHC	0.5188689	0	0	positive	P30
HALLMARK_MYC_TARGETS_V2	WDR4	LIHC	0.5785603	0	0	positive	P30
HALLMARK_MYC_TARGETS_V2	TRMT112	LIHC	0.5114798	0	0	positive	P30
HALLMARK_MYC_TARGETS_V2	METTL1	MESO	0.7155046	6.88E-15	3.44E-13	positive	P30
HALLMARK_MYC_TARGETS_V2	WDR4	MESO	0.5971162	1.03E-09	5.15E-08	positive	P30
HALLMARK_MYC_TARGETS_V2	METTL1	PCPG	0.5599614	4.44E-16	2.22E-14	positive	P30
HALLMARK_MYC_TARGETS_V2	METTL1	PRAD	0.6514047	0	0	positive	P30
HALLMARK_MYC_TARGETS_V2	WDR4	PRAD	0.5309274	0	0	positive	P30
HALLMARK_MYC_TARGETS_V2	METTL1	READ	0.5705767	8.88E-16	4.26E-14	positive	P30
HALLMARK_MYC_TARGETS_V2	WDR4	READ	0.5551041	8.44E-15	4.22E-13	positive	P30
HALLMARK_MYC_TARGETS_V2	WDR4	SARC	0.682664	0	0	positive	P30
HALLMARK_MYC_TARGETS_V2	METTL1	SKCM	0.5293938	8.96E-09	4.39E-07	positive	P30
HALLMARK_MYC_TARGETS_V2	WDR4	STAD	0.5100516	0	0	positive	P30

HALLMARK_MYC_TARGETS_V2	METTL1	TGCT	0.5353549	1.69E-12	7.76E-11	positive	P30
HALLMARK_MYC_TARGETS_V2	WBSCR22	TGCT	0.5084624	3.08E-11	1.20E-09	positive	P30
HALLMARK_MYC_TARGETS_V2	WDR4	TGCT	0.7182404	0.00E+00	0.00E+00	positive	P30
HALLMARK_MYC_TARGETS_V2	METTL1	THYM	0.530949	4.42E-10	2.21E-08	positive	P30
HALLMARK_MYC_TARGETS_V2	WBSCR22	THYM	0.6246295	2.46E-14	1.04E-12	positive	P30
HALLMARK_MYC_TARGETS_V2	WDR4	THYM	0.796246	0.00E+00	0.00E+00	positive	P30
HALLMARK_MYC_TARGETS_V2	WDR4	UCEC	0.513877	0	0	positive	P30
HALLMARK_MYC_TARGETS_V2	METTL1	UCS	0.513838	4.35E-05	0.002174768	positive	P30
HALLMARK_MYC_TARGETS_V2	METTL1	UVM	0.758115	4.44E-16	2.22E-14	positive	P30
HALLMARK_MYC_TARGETS_V2	WDR4	UVM	0.6066363	2.46E-09	1.20E-07	positive	P30
HALLMARK_MYC_TARGETS_V2	WDR4	BRCA	0.6127029	0	0	positive	P30
HALLMARK_MYC_TARGETS_V2	WDR4	CESC	0.5769195	0	0	positive	P30
HALLMARK_MYOGENESIS	RNMT	SKCM	-0.5105481	3.60E-08	1.73E-06	negative	N31
HALLMARK_MYOGENESIS	WBSCR22	TGCT	-0.5907901	1.78E-15	7.99E-14	negative	N31
HALLMARK_MYOGENESIS	FAM103A1	TGCT	-0.6963411	0.00E+00	0.00E+00	negative	N31
HALLMARK_MYOGENESIS	RNMT	UVM	-0.6920562	1.17E-12	5.72E-11	negative	N31
HALLMARK_NOTCH_SIGNALING	RNMT	LAML	-0.5025703	4.85E-11	2.09E-09	negative	N32
HALLMARK_NOTCH_SIGNALING	WBSCR22	TGCT	-0.5425705	7.40E-13	3.03E-11	negative	N32
HALLMARK_NOTCH_SIGNALING	FAM103A1	TGCT	-0.6924642	0.00E+00	0.00E+00	negative	N32
HALLMARK_NOTCH_SIGNALING	FAM103A1	UVM	-0.6659442	1.58E-11	7.89E-10	negative	N32
HALLMARK_OXIDATIVE_PHOSPHORYLATION	METTL1	BRCA	0.5196804	0	0	positive	P33
HALLMARK_OXIDATIVE_PHOSPHORYLATION	TRMT112	BRCA	0.527312	0	0	positive	P33
HALLMARK_OXIDATIVE_PHOSPHORYLATION	WBSCR22	BRCA	0.5203312	0	0	positive	P33
HALLMARK_OXIDATIVE_PHOSPHORYLATION	WBSCR22	CESC	0.5256538	0	0	positive	P33
HALLMARK_OXIDATIVE_PHOSPHORYLATION	METTL1	DLBC	0.5847491	1.27E-05	6.12E-04	positive	P33
HALLMARK_OXIDATIVE_PHOSPHORYLATION	WDR4	DLBC	0.6832438	8.75E-08	4.11E-06	positive	P33
HALLMARK_OXIDATIVE_PHOSPHORYLATION	TRMT112	DLBC	0.6638174	2.70E-07	1.30E-05	positive	P33
HALLMARK_OXIDATIVE_PHOSPHORYLATION	WBSCR22	DLBC	0.7317707	3.45E-09	1.65E-07	positive	P33
HALLMARK_OXIDATIVE_PHOSPHORYLATION	TRMT112	GBM	0.6850935	0.00E+00	0.00E+00	positive	P33
HALLMARK_OXIDATIVE_PHOSPHORYLATION	FAM103A1	GBM	0.542605	2.13E-13	1.04E-11	positive	P33
HALLMARK_OXIDATIVE_PHOSPHORYLATION	TRMT112	HNSC	0.5114794	0	0	positive	P33
HALLMARK_OXIDATIVE_PHOSPHORYLATION	WBSCR22	HNSC	0.5356904	0	0	positive	P33
HALLMARK_OXIDATIVE_PHOSPHORYLATION	RNMT	LIHC	-0.5106903	0	0	negative	N33
HALLMARK_OXIDATIVE_PHOSPHORYLATION	TRMT112	LUAD	0.5672137	0	0	positive	P33
HALLMARK_OXIDATIVE_PHOSPHORYLATION	FAM103A1	LUAD	0.52767	0	0	positive	P33
HALLMARK_OXIDATIVE_PHOSPHORYLATION	TRMT112	LUSC	0.5451004	0	0	positive	P33
HALLMARK_OXIDATIVE_PHOSPHORYLATION	TRMT112	MESO	0.518252	2.73E-07	1.31E-05	positive	P33
HALLMARK_OXIDATIVE_PHOSPHORYLATION	TRMT112	OV	0.6177621	0	0	positive	P33
HALLMARK_OXIDATIVE_PHOSPHORYLATION	METTL1	PRAD	0.5704842	0	0	positive	P33
HALLMARK_OXIDATIVE_PHOSPHORYLATION	WBSCR22	PRAD	0.6034711	0	0	positive	P33
HALLMARK_OXIDATIVE_PHOSPHORYLATION	RNMT	PRAD	-0.5321641	0	0	negative	N33
HALLMARK_OXIDATIVE_PHOSPHORYLATION	TRMT112	PRAD	0.6212401	0	0	positive	P33
HALLMARK_OXIDATIVE_PHOSPHORYLATION	WBSCR22	SARC	0.5158265	0	0	positive	P33
HALLMARK_OXIDATIVE_PHOSPHORYLATION	TRMT112	SARC	0.5249419	0	0	positive	P33
HALLMARK_OXIDATIVE_PHOSPHORYLATION	WDR4	TGCT	0.5526083	2.27E-13	9.78E-12	positive	P33
HALLMARK_OXIDATIVE_PHOSPHORYLATION	TRMT112	TGCT	0.5581187	1.17E-13	5.85E-12	positive	P33
HALLMARK_OXIDATIVE_PHOSPHORYLATION	RNMT	THCA	-0.5309489	0	0	negative	N33
HALLMARK_OXIDATIVE_PHOSPHORYLATION	WBSCR22	THYM	0.7555008	0.00E+00	0.00E+00	positive	P33
HALLMARK_OXIDATIVE_PHOSPHORYLATION	WDR4	THYM	0.5685562	1.25E-11	5.51E-10	positive	P33
HALLMARK_OXIDATIVE_PHOSPHORYLATION	TRMT112	THYM	0.5392315	2.10E-10	8.80E-09	positive	P33
HALLMARK_OXIDATIVE_PHOSPHORYLATION	WBSCR22	UVM	0.5140494	1.08E-06	5.38E-05	positive	P33
HALLMARK_P53_PATHWAY	RNMT	LAML	-0.5332106	1.81E-12	9.05E-11	negative	N34
HALLMARK_P53_PATHWAY	RNMT	SKCM	-0.5031284	6.08E-08	2.80E-06	negative	N34
HALLMARK_P53_PATHWAY	RNMT	UVM	-0.5113513	1.25E-06	4.88E-05	negative	N34
HALLMARK_PI3K_AKT_MTOR_SIGNALING	RNMT	LAML	-0.5194039	8.30E-12	3.73E-10	negative	N37
HALLMARK_PROTEIN_SECRETION	TRMT112	ACC	-0.5200414	9.02E-07	4.51E-05	negative	N38
HALLMARK_PROTEIN_SECRETION	TRMT112	DLBC	-0.5444986	6.31E-05	2.84E-03	negative	N38
HALLMARK_PROTEIN_SECRETION	WBSCR22	DLBC	-0.6446447	7.62E-07	3.43E-05	negative	N38
HALLMARK_PROTEIN_SECRETION	WBSCR22	KIRP	-0.6105967	0	0	negative	N38
HALLMARK_PROTEIN_SECRETION	RNMT	SKCM	0.6223016	2.27E-12	1.13E-10	positive	P38
HALLMARK_PROTEIN_SECRETION	TRMT112	SKCM	-0.5439548	2.88E-09	1.41E-07	negative	N38
HALLMARK_PROTEIN_SECRETION	WBSCR22	THYM	-0.7624026	0.00E+00	0.00E+00	negative	N38
HALLMARK_PROTEIN_SECRETION	RNMT	THYM	0.6912153	0.00E+00	0.00E+00	positive	P38
HALLMARK_PROTEIN_SECRETION	WDR4	THYM	-0.5873175	1.78E-12	8.18E-11	negative	N38
HALLMARK_PROTEIN_SECRETION	TRMT112	THYM	-0.7068473	0.00E+00	0.00E+00	negative	N38
HALLMARK_PROTEIN_SECRETION	RNMT	UVM	0.5823482	1.46E-08	6.56E-07	positive	P38
HALLMARK_PROTEIN_SECRETION	TRMT112	UVM	-0.6827069	3.06E-12	1.50E-10	negative	N38

HALLMARK_REACTIVE_OXYGEN_SPECIES_PATHWAY	METTL1	DLBC	0.6673488	2.22E-07	1.11E-05	positive	P39
HALLMARK_REACTIVE_OXYGEN_SPECIES_PATHWAY	METTL1	KIRC	0.5050417	0	0	positive	P39
HALLMARK_REACTIVE_OXYGEN_SPECIES_PATHWAY	RNMT	LAML	-0.524058	5.00E-12	2.40E-10	negative	N39
HALLMARK_REACTIVE_OXYGEN_SPECIES_PATHWAY	RNMT	LIHC	-0.5070782	0	0	negative	N39
HALLMARK_REACTIVE_OXYGEN_SPECIES_PATHWAY	RNMT	OV	-0.5003679	0	0	negative	N39
HALLMARK_REACTIVE_OXYGEN_SPECIES_PATHWAY	RNMT	THCA	-0.5109442	0	0	negative	N39
HALLMARK_REACTIVE_OXYGEN_SPECIES_PATHWAY	WBSCR22	THYM	0.6017249	3.64E-13	1.46E-11	positive	P39
HALLMARK_REACTIVE_OXYGEN_SPECIES_PATHWAY	WDR4	THYM	0.5759016	5.92E-12	2.66E-10	positive	P39
HALLMARK_REACTIVE_OXYGEN_SPECIES_PATHWAY	TRMT112	THYM	0.5055879	3.87E-09	1.55E-07	positive	P39
HALLMARK_REACTIVE_OXYGEN_SPECIES_PATHWAY	RNMT	UCS	-0.555849	7.17E-06	0.000358365	negative	N39
HALLMARK_REACTIVE_OXYGEN_SPECIES_PATHWAY	METTL1	UVM	0.5313848	3.95E-07	1.86E-05	positive	P39
HALLMARK_REACTIVE_OXYGEN_SPECIES_PATHWAY	RNMT	UVM	-0.5483232	1.40E-07	5.75E-06	negative	N39
HALLMARK_REACTIVE_OXYGEN_SPECIES_PATHWAY	WDR4	UVM	0.5525382	1.07E-07	5.04E-06	positive	P39
HALLMARK_SPERMATOGENESIS	RNMT	SKCM	0.5080055	4.31E-08	2.03E-06	positive	P40
HALLMARK_SPERMATOGENESIS	WBSCR22	TGCT	0.5843143	4.22E-15	1.77E-13	positive	P40
HALLMARK_SPERMATOGENESIS	WDR4	TGCT	0.5094646	2.78E-11	1.17E-09	positive	P40
HALLMARK_SPERMATOGENESIS	FAM103A1	TGCT	0.5590484	1.04E-13	4.07E-12	positive	P40
HALLMARK_SPERMATOGENESIS	RNMT	UVM	0.5667089	4.26E-08	1.83E-06	positive	P40
HALLMARK_TGF_BETA_SIGNALING	WBSCR22	DLBC	-0.6740044	1.51E-07	6.95E-06	negative	N41
HALLMARK_TGF_BETA_SIGNALING	WBSCR22	HNSC	-0.5396001	0	0	negative	N41
HALLMARK_TGF_BETA_SIGNALING	METTL1	LAML	-0.5607594	6.99E-14	3.36E-12	negative	N41
HALLMARK_TGF_BETA_SIGNALING	TRMT112	MESO	-0.5559748	2.27E-08	1.11E-06	negative	N41
HALLMARK_TGF_BETA_SIGNALING	METTL1	PRAD	-0.5125799	0	0	negative	N41
HALLMARK_TGF_BETA_SIGNALING	WBSCR22	PRAD	-0.5196455	0	0	negative	N41
HALLMARK_TGF_BETA_SIGNALING	METTL1	TGCT	-0.500645	6.84E-11	2.94E-09	negative	N41
HALLMARK_TGF_BETA_SIGNALING	WBSCR22	TGCT	-0.587185	2.89E-15	1.27E-13	negative	N41
HALLMARK_TGF_BETA_SIGNALING	RNMT	TGCT	0.5501335	3.05E-13	1.53E-11	positive	P41
HALLMARK_TGF_BETA_SIGNALING	FAM103A1	TGCT	-0.5411247	8.74E-13	3.06E-11	negative	N41
HALLMARK_TGF_BETA_SIGNALING	WBSCR22	THCA	-0.5175908	0	0	negative	N41
HALLMARK_TGF_BETA_SIGNALING	WBSCR22	THYM	-0.7520287	0.00E+00	0.00E+00	negative	N41
HALLMARK_TGF_BETA_SIGNALING	RNMT	THYM	0.5508118	7.12E-11	3.20E-09	positive	P41
HALLMARK_TGF_BETA_SIGNALING	TRMT112	THYM	-0.5239813	8.17E-10	3.35E-08	negative	N41
HALLMARK_TGF_BETA_SIGNALING	TRMT112	UCS	-0.5223644	3.07E-05	1.41E-03	negative	N41
HALLMARK_TNFA_SIGNALING_VIA_NFKB	WBSCR22	DLBC	-0.5091302	2.19E-04	8.12E-03	negative	N42
HALLMARK_UNFOLDED_PROTEIN_RESPONSE	WDR4	TGCT	0.7244225	0.00E+00	0.00E+00	positive	P43
HALLMARK_UNFOLDED_PROTEIN_RESPONSE	WDR4	UVM	0.6307951	3.58E-10	1.79E-08	positive	P43
HALLMARK_UV_RESPONSE_DN	WBSCR22	BLCA	-0.5560073	0.00E+00	0.00E+00	negative	N44
HALLMARK_UV_RESPONSE_DN	TRMT112	BRCA	-0.5081407	0	0	positive	P44
HALLMARK_UV_RESPONSE_DN	WBSCR22	BRCA	-0.5848543	0	0	negative	N44
HALLMARK_UV_RESPONSE_DN	METTL1	COAD	-0.5969044	0	0	negative	N44
HALLMARK_UV_RESPONSE_DN	WDR4	DLBC	-0.5113596	2.04E-04	8.96E-03	negative	N44
HALLMARK_UV_RESPONSE_DN	TRMT112	DLBC	-0.6922672	5.03E-08	2.51E-06	negative	N44
HALLMARK_UV_RESPONSE_DN	WBSCR22	DLBC	-0.7765949	8.75E-11	4.37E-09	negative	N44
HALLMARK_UV_RESPONSE_DN	TRMT112	GBM	-0.5528797	6.00E-14	2.70E-12	negative	N44
HALLMARK_UV_RESPONSE_DN	WBSCR22	HNSC	-0.5721891	0	0	negative	N44
HALLMARK_UV_RESPONSE_DN	WBSCR22	KICH	-0.5269841	5.47E-06	2.63E-04	negative	N44
HALLMARK_UV_RESPONSE_DN	TRMT112	KIRC	-0.559057	0	0	negative	N44
HALLMARK_UV_RESPONSE_DN	WBSCR22	KIRC	-0.6307294	0	0	negative	N44
HALLMARK_UV_RESPONSE_DN	METTL1	KIRP	-0.541897	0	0	negative	N44
HALLMARK_UV_RESPONSE_DN	RNMT	KIRP	0.5075741	0	0	positive	P44
HALLMARK_UV_RESPONSE_DN	TRMT112	KIRP	-0.5273692	0	0	negative	N44
HALLMARK_UV_RESPONSE_DN	WBSCR22	KIRP	-0.6390272	0	0	negative	N44
HALLMARK_UV_RESPONSE_DN	METTL1	LAML	-0.5002727	6.13E-11	2.64E-09	negative	N44
HALLMARK_UV_RESPONSE_DN	WBSCR22	LGG	-0.6560591	0	0	negative	N44
HALLMARK_UV_RESPONSE_DN	METTL1	LIHC	-0.5752431	0	0	negative	N44
HALLMARK_UV_RESPONSE_DN	WBSCR22	LIHC	-0.5452064	0	0	negative	N44
HALLMARK_UV_RESPONSE_DN	TRMT112	LIHC	-0.660112	0	0	negative	N44
HALLMARK_UV_RESPONSE_DN	WBSCR22	LUAD	-0.5118408	0	0	negative	N44
HALLMARK_UV_RESPONSE_DN	TRMT112	MESO	-0.6564387	5.10E-12	2.55E-10	negative	N44
HALLMARK_UV_RESPONSE_DN	METTL1	OV	-0.5208718	0	0	negative	N44
HALLMARK_UV_RESPONSE_DN	WBSCR22	PAAD	-0.6854843	0.00E+00	0.00E+00	negative	N44
HALLMARK_UV_RESPONSE_DN	TRMT112	PCPG	-0.6662147	0.00E+00	0.00E+00	negative	N44
HALLMARK_UV_RESPONSE_DN	METTL1	PRAD	-0.6570708	0	0	negative	N44
HALLMARK_UV_RESPONSE_DN	WBSCR22	PRAD	-0.6770263	0	0	negative	N44
HALLMARK_UV_RESPONSE_DN	RNMT	PRAD	0.5099488	0	0	positive	P44
HALLMARK_UV_RESPONSE_DN	TRMT112	PRAD	-0.6408548	0	0	negative	N44
HALLMARK_UV_RESPONSE_DN	METTL1	READ	-0.6057009	0.00E+00	0.00E+00	negative	N44

HALLMARK_UV_RESPONSE_DN	WBSCR22	SARC	-0.5502483	0	0	negative	N44
HALLMARK_UV_RESPONSE_DN	TRMT112	SARC	-0.5415284	0	0	negative	N44
HALLMARK_UV_RESPONSE_DN	METTL1	SKCM	-0.5424502	3.25E-09	1.62E-07	negative	N44
HALLMARK_UV_RESPONSE_DN	TRMT112	SKCM	-0.5095686	3.86E-08	1.81E-06	negative	N44
HALLMARK_UV_RESPONSE_DN	WBSCR22	STAD	-0.5326336	0	0	negative	N44
HALLMARK_UV_RESPONSE_DN	METTL1	TGCT	-0.7146348	0.00E+00	0.00E+00	negative	N44
HALLMARK_UV_RESPONSE_DN	WBSCR22	TGCT	-0.7144877	0.00E+00	0.00E+00	negative	N44
HALLMARK_UV_RESPONSE_DN	TRMT112	TGCT	-0.5442261	6.11E-13	2.93E-11	negative	N44
HALLMARK_UV_RESPONSE_DN	FAM103A1	TGCT	-0.6408513	0.00E+00	0.00E+00	negative	N44
HALLMARK_UV_RESPONSE_DN	WBSCR22	THCA	-0.6920675	0	0	negative	N44
HALLMARK_UV_RESPONSE_DN	RNMT	THCA	0.5454025	0	0	positive	P44
HALLMARK_UV_RESPONSE_DN	TRMT112	THCA	-0.6839696	0	0	negative	N44
HALLMARK_UV_RESPONSE_DN	WBSCR22	THYM	-0.7983976	0.00E+00	0.00E+00	negative	N44
HALLMARK_UV_RESPONSE_DN	RNMT	THYM	0.5889205	1.50E-12	7.03E-11	positive	P44
HALLMARK_UV_RESPONSE_DN	WDR4	THYM	-0.6115792	1.17E-13	5.51E-12	negative	N44
HALLMARK_UV_RESPONSE_DN	TRMT112	THYM	-0.6826907	0.00E+00	0.00E+00	negative	N44
HALLMARK_UV_RESPONSE_DN	METTL1	UCEC	-0.5200699	0	0	negative	N44
HALLMARK_UV_RESPONSE_DN	TRMT112	UCEC	-0.5679816	0	0	negative	N44
HALLMARK_UV_RESPONSE_DN	WBSCR22	UCS	-0.6426983	7.04E-08	3.52E-06	negative	N44
HALLMARK_UV_RESPONSE_DN	TRMT112	UCS	-0.5954676	1.03E-06	5.15E-05	negative	N44
HALLMARK_UV_RESPONSE_DN	TRMT112	UVM	-0.6013199	3.68E-09	1.69E-07	negative	N44
HALLMARK_UV_RESPONSE_UP	METTL1	DLBC	0.5030547	2.68E-04	1.18E-02	positive	P45
HALLMARK_UV_RESPONSE_UP	METTL1	KIRC	0.5134511	0	0	positive	P45
HALLMARK_UV_RESPONSE_UP	RNMT	LAML	-0.5331183	1.83E-12	9.05E-11	negative	N45
HALLMARK_UV_RESPONSE_UP	METTL1	UVM	0.6645757	1.80E-11	8.62E-10	positive	P45
HALLMARK_UV_RESPONSE_UP	RNMT	UVM	-0.7253353	2.75E-14	1.38E-12	negative	N45
HALLMARK_UV_RESPONSE_UP	WDR4	UVM	0.5138012	1.09E-06	4.91E-05	positive	P45
HALLMARK_UV_RESPONSE_UP	TRMT112	UVM	0.5091548	1.42E-06	6.23E-05	positive	P45
HALLMARK_WNT_BETA_CATENIN_SIGNALING	WBSCR22	DLBC	-0.5817471	1.45E-05	6.22E-04	negative	N46
HALLMARK_WNT_BETA_CATENIN_SIGNALING	WBSCR22	THYM	-0.6235697	2.80E-14	1.15E-12	negative	N46
HALLMARK_XENOBIOTIC_METABOLISM	RNMT	LAML	-0.5011192	5.63E-11	2.36E-09	negative	N47
HALLMARK_XENOBIOTIC_METABOLISM	RNMT	UVM	-0.5441161	1.82E-07	7.29E-06	negative	N47
